# Federally Qualified Health Centers and Performance of Medicare Accountable Care Organizations

**DOI:** 10.1001/jamanetworkopen.2024.45536

**Published:** 2024-11-18

**Authors:** Kun Li, Yucheng Hou, Frank McStay, Jonathan Gonzalez-Smith, Robert S. Saunders

**Affiliations:** 1 Duke-Margolis Institute for Health Policy, Duke University, Washington, DC; 2 The University of Texas Health Science Center at Houston

## Abstract

**Importance:**

Federally qualified health centers (FQHCs) have increasingly participated in the Medicare Shared Savings Program (MSSP) accountable care organizations (ACOs), one of the most widespread value-based programs. Although FQHCs may strengthen ACOs’ ability to provide affordable care to diverse Medicare beneficiaries, evidence on ACOs’ performance by FQHC participation is limited.

**Objectives:**

To compare beneficiary characteristics, utilization, expenditure, and quality between ACOs with and without FQHC participation and assess changes in ACO performance after including first FQHCs.

**Design, Setting, and Participants:**

Using MSSP public use files, this cross-sectional study compared performance of ACOs that always had FQHC participation with ACOs that never had FQHC participation from January 1, 2016, to December 31, 2022, supplemented with staggered difference-in-differences analyses of ACOs’ first-time inclusion of FQHCs on performance measures. Data analysis was performed from December 1, 2023, to February 29, 2024.

**Exposure:**

Participation of FQHCs in the MSSP.

**Main Outcomes and Measures:**

Measures of ACO-assigned beneficiaries, utilization, expenditure, and quality per ACO-year.

**Results:**

Among 752 ACOs in the descriptive analysis, 140 ACOs always had at least 1 FQHC participant, whereas 612 ACOs never had FQHC participants. Compared with ACOs that never had FQHC participation, those that always had FQHC participation provided care to more socioeconomically disadvantaged beneficiaries (mean [SD] with dual eligibility, 2035.8 [2110.6] vs 1040.9 [1084.2] person-years; with disability, 3341.1 [3474.9] vs 1705.1 [1664.9] person-years; in racial and ethnic minoritized groups, 3690.6 [4118.4] vs 2515.1 [2762.9] person-years), with fewer primary care visits (mean [SD], 9956.6 [1926.3] vs 10 858.8 [2383.4] per 1000 person-years), more emergency department visits (mean [SD], 771.6 [190.9] vs 657.2 [160.0] per 1000 person-years), and lower levels of several quality measures. In the difference-in-differences analysis, 43 ACOs included FQHCs for the first time. Including first FQHCs was associated with increases of 872.9 dual-eligible (95% CI, 345.9-1399.8), 1137.6 disability (95% CI, 390.1-1885.1), and 1350.8 racial and ethnic minority (95% CI, 447.4-2254.1) person-years, with increases in rates of influenza immunization (5.9 percentage points [pp]; 95% CI, 1.4-10.4 pp), tobacco screening and cessation intervention (11.8 pp; 95% CI, 3.7-20.0 pp), and depression screening and follow-up (8.9 pp; 95% CI, 0.5-17.4 pp). No associations were observed between FQHC inclusion and utilization or expenditure.

**Conclusions and Relevance:**

In this repeated cross-sectional study, MSSP ACOs with FQHC participation served more socioeconomically disadvantaged Medicare beneficiaries than those without FQHC participation. The inclusion of first FQHCs was associated with increased rates of several preventive services without increasing costs. Participation of safety net practices appeared to improve access to ACOs among beneficiaries from underserved communities.

## Key Points

**Question:**

Is the participation of federally qualified health centers (FQHCs) in Medicare Shared Savings Program accountable care organizations (ACOs) associated with ACOs’ beneficiary characteristics, utilization, expenditure, and quality?

**Findings:**

In this repeated cross-sectional study, after including FQHCs for the first time, ACOs served more beneficiaries who were low income, with disabilities, or who were members of racial and ethnic minoritized groups, with increased levels of several quality measures associated with the delivery of preventive care compared with ACOs without FQHC participation.

**Meaning:**

Participation of FQHCs in Medicare Shared Savings Program ACOs may enhance reach to accountable care among socioeconomically disadvantaged populations, without increasing expenditure or reducing quality.

## Teaser

This cross-sectional study compared beneficiary characteristics, utilization, expenditure, and quality between accountable care organizations (ACOs) with and without federally qualified health center (FQHC) participation and assessed changes in ACO performance after including first FQHCs.

## Introduction

Engaging safety net practices in ongoing payment and delivery system reform is critical to ensure equitable access to high-value care.^1,2^ Serving more than 30 million individuals regardless of their ability to pay,^3^ federally qualified health centers (FQHCs) are a natural policy target to be included in numerous value-based payment models,^4,5,6^ especially those with a population health and primary care focus. In particular, the participation of FQHCs in accountable care organizations (ACOs) under the Medicare Shared Savings Program (MSSP) has increased markedly, from 60 delivery sites in 2016 to more than 4000 in 2023.^7^

An ACO is a population-based, total cost of care model in which a group of practices voluntarily contract to meet the quality standards and cost benchmarks for their attributed beneficiaries in return for shared savings or losses.^8^ With the strength in providing culturally aware primary care, FQHCs could be well positioned to care for socially and medically underserved Medicare beneficiaries attributed to ACOs.^3,9,10^ FQHCs also align with ACO incentives to provide high-quality care at lower costs.^3,11,12,13,14^ However, FQHCs often face financial constraints and inadequate infrastructure investment (eg, electronic health record capacity).^15,16^ Moreover, their patients usually have complex health and social needs, limited access to specialty care, and higher utilization of emergency departments (EDs) compared with non–safety net practices.^17,18^ These features may restrict ACOs’ ability to generate savings and deter the inclusion of FQHCs in their participant lists.

Despite the significant increase in FQHC participation, evidence on the association between FQHC participation and ACO performance is lacking. Previous ACO evaluations predominantly focused on comparing spending and utilization between ACO-attributed and non–ACO-attributed beneficiaries.^6,19,20,21,22^ Several studies have compared hospital-led with physician-led ACOs.^21,23,24^ Less attention has been paid to examining ACO performance by other sets of practice compositions, including FQHCs. A qualitative study of 173 ACOs during the early years of MSSP (2012-2013) suggests that ACOs with community health centers (including FQHCs) are similar to other ACOs in organizational structure and capabilities regarding care management and quality improvement.^25^ A recent descriptive analysis found that MSSP ACOs with more FQHC participants were more likely to generate shared savings in 2022.^26^ Much of this evidence, however, relied on information from a single point in time; implications for beneficiary compositions and quality of care were not examined. This study provides new data on whether the participation of FQHCs in MSSP ACOs is associated with changes in ACO beneficiary characteristics, utilization, expenditure, and quality.

## Methods

This repeated cross-sectional study used information on all MSSP ACOs from January 1, 2016, to December 31, 2022, a period that covers most of the major policy changes to the MSSP, including the Pathways to Success.^27^ We first compared characteristics and performance of ACOs between those that always had FQHCs and those that never had FQHCs during the study period. Second, we examined annual changes in outcomes associated with the inclusion of first FQHCs in ACO contracts compared with ACOs that never had FQHC participation. Because of the unique patient panels of FQHCs, we hypothesized that the first-time inclusion of FQHCs was associated with increased reach to socioeconomically disadvantaged beneficiaries. Duke University did not require review of this study because all data were publicly available. Informed consent was also waived because the data did not identify individual patients. This study followed the Strengthening the Reporting of Observational Studies in Epidemiology (STROBE) reporting guideline.^28^

### Study Data and FQHC Participation

We used the 2016-2022 Shared Savings Program Performance Year Financial and Quality Results from MSSP public use files.^29^ These files include ACO-level information about characteristics of attributed beneficiaries, health services utilization, practice composition, expenditures, and quality of care in each performance year. We defined 2 types of FQHC participation. First, for the descriptive analysis, we defined ACOs that always had FQHC participation as those having at least 1 FQHC participant in all years when they appeared in the data. Second, for the difference-in-differences analysis, we defined ACOs that included FQHCs for the first time (treated group) as those that had no FQHC participation when they first appeared in the data and then included at least 1 FQHC participant. The control group included ACOs that never had FQHC participation until 2022.

### Sample

We included all ACO-years for the descriptive analysis. For the difference-in-differences analysis that examined the association of first-time inclusion of FQHCs and ACO-level outcomes, we included ACOs that appeared in the data for at least 2 years. We further excluded ACOs with no pre-FQHC participation data (always treated) and ACOs that included FQHCs but dropped all FQHCs in later years (switchers) (eTable 1 in Supplement 1). In sensitivity analyses, we tested the inclusion of additional ACO-years of switchers until they dropped all FQHCs.

### Outcome Variables

We analyzed the total number of person-years attributed to ACOs and the number of person-years by different demographic characteristics, including dual eligibility for Medicare and Medicaid, disability status, and race and ethnicity. The number of attributed person-years by race and ethnicity was recorded in the public use files based on beneficiaries’ most current Medicare records.^30^ We analyzed racial and ethnic minoritized person-years collectively, categorized as American Indian or Alaska Native, Asian and Pacific Islander, Black, Hispanic, other (no additional information available for this group from the Centers for Medicare & Medicaid Services data), and unknown, due to the high frequency of Centers for Medicare & Medicaid Services data suppression for services delivered to 10 or fewer beneficiaries.

We examined per-capita expenditure and 5 utilization outcomes at the ACO level, including the number of FQHC and rural health clinic (RHC) visits, total primary care visits, outpatient ED visits, short-term acute care hospital discharges, and skilled nursing facility (SNF) discharges, all measured in 1000 person-years. We imputed the missing values with zero for outcomes of beneficiary person-year, utilization, and expenditure. In sensitivity analysis, we log-transformed these outcomes and excluded observations with zero counts because distributions of these variables are usually highly skewed. We also analyzed 8 quality measures, defined as the percentage of eligible beneficiaries who received certain types of preventive care services (influenza immunization, tobacco use screening and cessation intervention, screening for depression and follow-up plan, colorectal cancer screening, and breast cancer screening) or achieved certain outcomes (depression remission at 12 months, patients with diabetes with a controlled glycated hemoglobin level, and patients with hypertension but controlled blood pressure).

### Statistical Analysis

We first described trends of percentage of MSSP ACOs with FQHC participation. Second, we used a 2-tailed, independent-sample *t* test to compare unadjusted means of characteristics among ACO-years that always had FQHC participation with those that never had FQHC participation during the study period. To further examine the changes in outcomes after the first-time inclusion of FQHCs, we used event-study difference-in-differences approaches with staggered treatment timing. This approach allows us to compare outcomes among ACOs that included first FQHCs (treated ACOs) with those that never included any FQHCs (control ACOs), before and after the inclusion, under the assumption that the treated ACOs would have experienced a similar trend in outcomes as the control ACOs in the absence of FQHC inclusion. The number of ACOs that included first FQHCs by year is given in eTable 2 in Supplement 1. We used the Callaway and Sant’Anna method to account for potential heterogeneous treatment effects across ACOs that included FQHCs at varying periods.^31^ We estimated differential changes in outcomes among treated ACOs and control ACOs over time as well as aggregated changes across 4 years after the FQHC inclusion. For each regression, we empirically tested the parallel trend assumption up to 3 years before the FQHC inclusion. Detailed empirical approaches are given in the eMethods in Supplement 1.

We computed 95% CIs based on SEs clustered at the ACO level to account for repeated observations within an ACO. A 2-sided significance threshold was set at *P* < .05. We evaluated the robustness of our results to the sharpened 2-stage q values that account for multiple comparisons.^32^ All statistical analyses were performed using Stata/SE, version 18.0 (StataCorp LLC). Data analysis was performed from December 1, 2023, to February 29, 2024.

## Results

### Trends in FQHC Participation in MSSP ACOs

**Figure 1.**
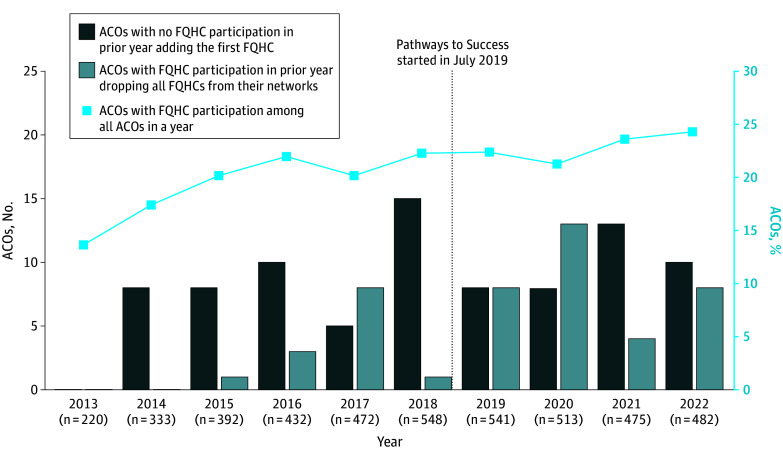
Trend in the Number of Medicare Shared Savings Program Accountable Care Organizations (ACOs) Adding and Dropping Federally Qualified Health Centers (FQHCs) and Percentage of ACOs With FQHC Participation

The percentage of MSSP ACOs with FQHC participation increased from 13.6% (30 of 220) in 2013 to 24.3% (117 of 482) in 2022 (Figure 1). After 2013, several ACOs with no FQHC participation in the prior year included at least 1 FQHC in their networks, ranging from 5 to 15 ACOs each year. Forty-six ACOs with FQHC participation in the prior year dropped all FQHCs from their networks during this period. More than half of dropouts happened during 2020 to 2022, a period likely affected by the COVID-19 pandemic and the implementation of the Pathways to Success that requires all ACOs to assume greater downside risks in an accelerated time frame.

### ACO Characteristics by FQHC Participation

**Figure 2.**
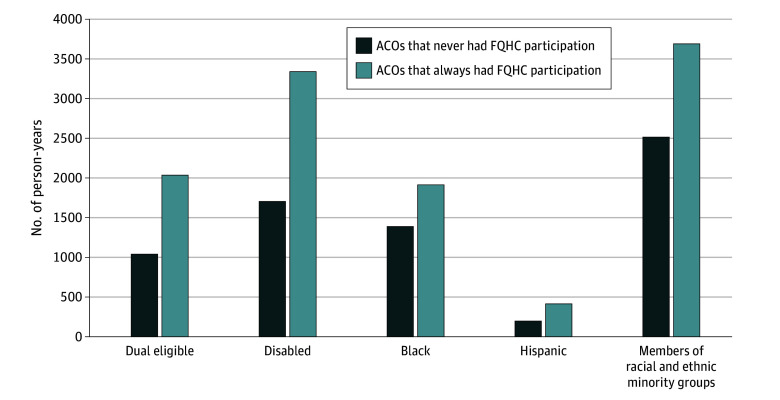
Characteristics of Beneficiaries Assigned to Medicare Shared Savings Program Accountable Care Organizations (ACOs) That Never Had Federally Qualified Health Center (FQHC) Participation vs Always Had FQHC Participation, 2016-2022 Each bar represents the mean number of beneficiaries among ACO-years that never had FQHC participation (2457 ACO-years from 612 ACOs) or ACO-years that always had FQHC participation (545 ACO-years from 140 ACOs) during 2016-2022. Racial and ethnic minority groups include all beneficiaries who are American Indian or Alaska Native, Asian and Pacific Islander, Black, Hispanic, other, and unknown.

During 2016 to 2022, a total of 140 ACOs always had at least 1 FQHC participant, whereas 612 ACOs never had an FQHC participant. Figure 2 depicts unadjusted mean person-years of Medicare beneficiaries assigned to an ACO by FQHC participation. Compared with ACO-years that never had FQHC participation, those that always had FQHC participation had higher mean (SD) person-years overall (23 109.0 [28 839.3] vs 17 793.6 [17 089.3]) and for those with dual eligibility (2035.8 [2110.6] vs 1040.9 [1084.2]), with disability (3341.1 [3474.9] vs 1705.1 [1654.9]), and who were members of racial and ethnic minoritized groups (3690.6 [4118.4] vs 2515.1 [2762.9]) (*P* < .001 for all) (Table 1).

**Table 1.** Characteristics of Medicare Shared Savings Program ACOs With and Without Participation of FQHCs, 2016-2022

Characteristic	Mean (SD)		Difference	*P* value^a^
	Never had FQHC participation (612 ACOs and 2457 ACO-years)	Always had FQHC participation (140 ACOs and 545 ACO-years)		
ACO-assigned beneficiaries, No. of person-years				
Total	17 793.6 (17 089.3)	23 109.0 (28 839.3)	5315.4	<.001
Dual eligible	1040.9 (1084.2)	2035.8 (2110.6)	994.9	<.001
Disability	1705.1 (1664.9)	3341.1 (3474.9)	1636.0	<.001
Non-Hispanic Black	1388.7 (1923.2)	1914.8 (2576.4)	526.1	<.001
Hispanic	197.8 (373.7)	413.8 (613.6)	216.0	<.001
Racial and ethnic minoritized^b^	2515.1 (2762.9)	3690.6 (4118.4)	1175.5	<.001
Utilization, No. per 1000 person-years				
FQHC and RHC visits	245.1 (709.1)	1643.4 (1970.6)	1398.3	<.001
Total primary care visits	10 858.8 (2383.4)	9956.6 (1926.3)	−902.2	<.001
Outpatient emergency department visits	657.2 (160.0)	771.6 (190.9)	114.5	<.001
Short-term acute care hospital discharges	272.3 (74.7)	276.8 (60.9)	4.5	.19
Skilled nursing facility discharges	60.8 (52.1)	56.6 (33.1)	−4.2	.07
Per-capita expenditure, $	11 212.3 (2647.7)	10 974.2 (1927.4)	−238.1	.047
Quality, %				
Influenza immunization	75.5 (11.8)	70.3 (11.9)	−5.2	<.001
Tobacco use screening and cessation intervention	82.1 (16.4)	80.9 (16.3)	−1.2	.13
Screening for depression and follow-up plan	68.1 (19.4)	66.1 (17.9)	−2.1	.02
Colorectal cancer screening	70.7 (12.6)	65.0 (12.4)	−5.7	<.001
Breast cancer screening	73.7 (11.0)	69.5 (10.7)	−4.3	<.001
Depression remission at 12 mo	12.1 (13.3)	10.4 (9.2)	−1.7	.01
Diabetes control (glycated hemoglobin ≤9%)	85.8 (7.9)	82.7 (8.1)	−3.1	<.001
Controlling high blood pressure	74.2 (8.2)	70.3 (7.8)	−3.8	<.001

Abbreviations: ACO, accountable care organization; FQHC, federally qualified health center; RHC, rural health clinic.

^a^ Two-sided *t* test.

^b^ Includes all beneficiaries who are American Indian or Alaska Native, Asian and Pacific Islander, Black, Hispanic, other (no additional information available for this group from the Centers for Medicare & Medicaid Services data), and unknown.

Compared with ACOs that never had FQHC participation, those that always had FQHC participation were more likely to have fewer primary care visits (mean [SD], 9956.6 [1926.3] vs 10 858.8 [2383.4]) and more outpatient ED visits (mean [SD], 771.6 [190.9] vs 657.2 [160.0]) per 1000 person-years (*P* < .001 for both); ACOs that always had FQHC participation had lower per-capita expenditure (mean [SD], $10 974.2 [$1927.4] vs $11 212.3 [$2647.7]; *P* = .047) (Table 1). No significant differences were observed in ACOs that always had FQHC participation vs those with no FQHC participation regarding mean (SD) short-term acute care hospital discharges (276.8 [60.9] vs 272.3 [74.7]; *P* = .19), and SNF discharges (56.6 [33.1] vs 60.8 [52.1]; *P* = .07). When examining quality of care, we observed that ACOs with FQHC participation performed worse than those without FQHC participation across 7 of 8 quality measures.

### Changes in ACO Performance After First-Time Inclusion of FQHCs

The difference-in-differences analyses included 540 ACOs (2565 ACO-year observations). eTable 3 in Supplement 1 lists the baseline ACO characteristics by FQHC inclusion. The ACOs that included first FQHCs during the study period were generally similar to control ACOs at baseline, except that they had a higher rate of tobacco use screening and cessation intervention. No significant outcome differences were found between the treated ACOs and control ACOs during the pre-FQHC inclusion period (Table 2; eFigure in Supplement 1).

**Table 2.** Estimated Changes in Medicare Shared Savings Program ACO-Assigned Beneficiaries, Utilization, and Expenditure Associated With Participation of FQHCs From Difference-in-Differences Analyses

Outcome	Baseline value^a^	Estimated change (95% CI)	*P* value	Pretrend *P* value^b^
Assigned beneficiaries, No. of person-years				
Total	16 818.1	9594.1 (2249.4 to 16 938.8)	.01	.68
Dual eligible	1095.5	872.9 (345.9 to 1399.8)	<.001	.35
Disabled	2107.1	1137.6 (390.1 to 1885.1)	<.001	.59
Racial and ethnic minoritized^c^	2241.9	1350.8 (447.4 to 2254.1)	<.001	.68
Utilization, No. per 1000 person-years				
FQHC and RHC visits	302.6	494.5 (254.0 to 735.0)	<.001	.99
Total primary care visits	10 391.9	325.8 (−143.4 to 795.0)	.17	.44
Outpatient emergency department visits	691.1	6.7 (−43.5 to 56.9)	.79	.64
Short-term acute care hospital discharges	282.4	10.4 (−3.6 to 24.4)	.15	.38
Skilled nursing facility discharges	59.1	5.0 (−0.6 to 10.6)	.08	>.99
Per-capita expenditure, $	10 559.5	169.0 (−157.6 to 495.5)	.31	.47
Quality, %				
Influenza immunization	71.9	5.9 (1.4 to 10.4)	.01	.44
Tobacco use screening and cessation intervention	76.8	11.8 (3.7 to 20.0)	.004	.73
Screening for depression and follow-up plan	63.4	8.9 (0.5 to 17.4)	.04	.87
Colorectal cancer screening	67.1	1.3 (−2.4 to 5.1)	.48	.79
Breast cancer screening	71.8	−0.8 (−3.8 to 2.1)	.57	.48
Depression remission at 12 mo	10.4	3.2 (−4.5 to 10.8)	.42	.89
Diabetes control (glycated hemoglobin ≤9%)	83.6	0.9 (−2.8 to 4.7)	.62	.07
Controlling high blood pressure	72.2	0.8 (−1.6 to 3.1)	.52	.65

Abbreviations: ACO, accountable care organization; FQHC, federally qualified health center; RHC, rural health clinic.

^a^ Means of outcomes across all pretreatment years among ACOs that included FQHCs during 2016 to 2022.

^b^ Pretrend tests report the *P* value from a joint F test that the estimated coefficients of up to 3 years before FQHC inclusions are equal and not statistically significant from zero.

^c^ Includes all beneficiaries who are American Indian or Alaska Native, Asian and Pacific Islander, Black, Hispanic, other (no additional information available for this group from the Centers for Medicare & Medicaid Services data), and unknown.

Table 2 presents estimated changes in ACO characteristics and performance after the ACOs’ first-time FQHC inclusion. A typical ACO had a mean (SD) 16 818.1 (16 036.6) total person-years, 1095.5 (1219.9) dual-eligible person-years, 2107.1 (1985.9) disabled person-years, and 2241.9 (2344.9) racial and ethnic minority person-years. Inclusion of FQHCs was associated with significant increases in total person-years by 9594.1 (95% CI, 2249.4-16938.8), dual-eligible person-years by 872.9 (95% CI, 345.9-1399.8), disability person-years by 1137.6 (390.1-1885.1), and racial and ethnic minoritized group person-years by 1350.8 (95% CI, 447.4-2254.1).

When examining utilization among ACO beneficiaries, first-time FQHC inclusion was associated with 494.5 more FQHC and RHC visits per 1000 person-years in a year (95% CI, 254.0-735.0) but was not associated with total primary care visits, outpatient ED visits, short-term acute care hospital discharges, and SNF discharges, measured per 1000 person-years. Similarly, no association was observed between FQHC inclusion and an ACO’s per-capita expenditure in a year.

First-time FQHC inclusion was associated with several process-based quality measures, including a 5.9–percentage point (pp) increase (95% CI, 1.4-10.4 pp) in influenza immunization, 11.8-pp increase (95% CI, 3.7-20.0 pp) in tobacco use screening and cessation intervention, and 8.9-pp increase (95% CI, 0.5-17.4 pp) in screening for depression and follow-up plan. Detailed regression results are given in eTable 4 in Supplement 1.

In sensitivity analyses, we obtained qualitatively similar results when using sharpened q values for multiple comparison adjustment (eTable 5 in Supplement 1), using log-transformed outcomes (eTable 6 in Supplement 1), and including switcher ACOs that later dropped all FQHCs (eTable 7 in Supplement 1). eTable 7 in Supplement 1 also reports results from the baseline 2-way fixed-effects model without the Callaway and Sant’Anna^31^ adjustment. We found no associations between FQHC inclusion and the percentage of dual-eligible, disability, and racial and ethnic minoritized group person-years (eTable 8 in Supplement 1).

## Discussion

This study provided new evidence of the association between FQHC participation and the performance of MSSP ACOs. The percentage of ACOs with FQHC participants increased over time. The participation rate persisted during 2019 to 2022, a period likely affected by the launch of Pathways to Success and the COVID-19 pandemic.

Compared with ACOs without FQHC participation, those that always had FQHCs were larger and had more attributed beneficiaries who were dually enrolled in Medicare and Medicaid, had disability, or were from racial and ethnic minoritized groups. These descriptive results were in concert with our findings from the difference-in-differences analysis. Including the first FQHCs in an ACO was associated with an increase in the total number of attributed person-years, especially that of socioeconomically disadvantaged beneficiaries, during the 4-year period after the FQHC inclusion compared with ACOs that never included any FQHCs. Our findings suggest that the inclusion of FQHCs could bring new patients to ACOs. The increase in newly attributed beneficiaries from FQHCs may enlarge the potential referral base and revenue sources of specialists and hospitals participating in the same ACO. In addition, ACOs could provide value-based accountable care relationships for Medicare FQHC users, a group historically lacking access to specialty care and care coordination.^9,12,25,33^

We found that ACOs that always had FQHC participation had fewer primary care visits and more ED visits among their attributed beneficiaries, consistent with the care patterns of Medicare FQHC users.^17^ Additionally, ACOs that always had FQHC participation performed worse across 7 of 8 quality measures related to preventive and primary care. The observed unadjusted differences may be because FQHC users are more likely to have multiple chronic conditions and other complex needs,^34^ resulting in worse outcomes. However, when comparing ACOs with similar baseline characteristics, we found that first-time inclusion of FQHCs was not associated with changes in the utilization of primary care, ED, acute care hospitals, and SNFs. Consequently, no associations were observed between FQHC inclusion and per-capita expenditures. Moreover, first-time inclusion of FQHCs was associated with increases in rates of delivering several preventive services, including influenza immunization, tobacco use screening, and depression screening, consistent with the literature that FQHCs provide more preventive services than other types of primary care practices.^3,11,12,13^

Besides that the inclusion of FQHCs tends to improve ACO performance, participation in ACOs may also bring benefits to FQHCs. For example, ACOs could enhance FQHC users’ access to specialty care through referrals, especially those with hospitals and health systems.^35^ Although Medicare beneficiaries only account for 10% of FQHC patients,^3^ participating in MSSP ACOs would give FQHCs access to capital for infrastructure investment and clinical transformations,^36^ which will benefit not only Medicare and dual-eligible beneficiaries but also all patients served by FQHCs. Future studies could examine the potential implications of ACO participation on FQHCs.

Despite the mutually beneficial relationship between ACOs and FQHCs, mounting evidence suggests that practices often serving members of racial or ethnic minoritized groups and complex populations are less likely to participate in and more likely to exit ACO programs.^37,38^ Specifically, only a quarter of FQHC delivery sites participated in the MSSP in 2022.^26^ Barriers for safety net practices to participate in value-based payments often include organizational and funding fragmentation, poor financial viability to bear downside risks, inadequate risk adjustment, and lack of resources.^36,39^ To date, low-income and racial or ethnic minoritized beneficiaries are still underrepresented in Medicare value-based payment models, the performance of which will ultimately depend on aligned incentives and partnerships among all participating practices.^40^ With the progress toward advancing health equity through payment reform,^41^ several policy designs have been launched to accelerate the participation of safety net practices in value-based payments. For example, Pathways to Success includes additional financial incentives for safety net practices in MSSP, and a recent study found that ACOs with FQHCs, RHCs, or critical access hospitals were more likely to stay in the MSSP after its implementation.^42^ Other programs, such as the Making Care Primary and ACO REACH (Realizing Equity, Access, and Community Health), proposed up-front infrastructure investment and health equity benchmark adjustment to support care transformations and assume downside risks.^43,44^ Our study highlights the potential implication for access and quality of care among socioeconomically disadvantaged Medicare beneficiaries when these incentives to encourage safety net participation are in place.

### Limitations

The study has several limitations. First, our results are relevant to MSSP ACOs and may not be generalizable to other ACO programs. However, a detailed analysis of the largest ACO program that existed in the US for more than a decade allows for the unique investigation of the association between FQHC participation and ACO performance over time and across a nationwide sample of ACOs, which can be informative for other ACO programs in Medicare, Medicaid, and commercial plans. Second, our study is limited by only examining performance measures at the ACO level. Future studies with patient-level data are needed to understand how FQHC participation affects care processes and outcomes of FQHC patients who are newly attributed to an ACO. Third, our study focused on changes in ACO performance associated with binary FQHC participation (yes vs no). Future research could explore changes in ACO performance associated with the number of FQHC participants. Fourth, our difference-in-differences analyses are not causal. The decision to include FQHCs may be associated with unobserved ACO characteristics. Including FQHCs may also coincide with the overall expansion of ACOs. An ACO may include other types of health care practices at the same time it includes the first FQHCs, although we found that the number of RHCs and critical access hospitals did not change after the FQHC inclusion (eTable 9 in Supplement 1). Fifth, our study only examined FQHC participation and may not be generalized to other types of practices. Because regulatory requirements and financial incentives vary by practice type, participation of other practice types may impact ACO performance differently. Future studies are recommended to explore other types of practices, including RHCs and critical access hospitals.

## Conclusions

In this repeated cross-sectional study, MSSP ACOs that always had FQHC participation were larger and served more beneficiaries who were dually eligible for Medicare and Medicaid, who had disabilities, or who were members of racial or ethnic minoritized groups compared with ACOs that had no FQHC participation. Including the first FQHCs in ACO networks was associated with not only increased reach to socioeconomically disadvantaged beneficiaries but also increased levels of several quality measures at ACOs, with no changes in expenditure or utilization.

## Notes

Supplement 1. **eMethods.** Empirical Approaches
eReferences.
**eTable 1.** Sample Inclusion and Exclusion Criteria for the Difference-in-Difference Analysis **eTable 2.** Timing of ACOs Including FQHCs for the First Time, 2017-2022 **eTable 3.** Baseline Characteristics of MSSP ACOs in the Analytic Sample for the Difference-in-Difference Analysis **eTable 4.** Estimated Change in MSSP ACO-Assigned Beneficiaries, Utilization, Expenditure, and Quality Measures Associated With Participation of Federally Qualified Health Centers **eTable 5.** Comparison of Unadjusted p-values and Sharpened False Discovery Rate q-values for Multiple Comparison Adjustment **eTable 6.** Sensitivity Analysis: Estimated Percent Change in MSSP ACO-Assigned Beneficiaries, Utilization, and Expenditure Associated With Participation of Federally Qualified Health Centers using Logged Dependent Variables **eTable 7.** Sensitivity Analysis: Estimated Change in MSSP ACO-Assigned Beneficiaries, Utilization, Expenditure, and Quality Measures Associated With Participation of Federally Qualified Health Centers Using Alternative Specifications **eTable 8.** Sensitivity Analysis: Estimated Percentage-Point Change in MSSP ACO-Assigned Beneficiaries Associated With Participation of Federally Qualified Health Centers **eTable 9.** Sensitivity Analysis: Estimated Change in MSSP ACO Provider Compositions Associated With Participation of Federally Qualified Health Centers **eFigure.** Event Study Plots of Difference-in-Difference Analyses for Trends in Outcomes Among Treated and Control ACOs

Supplement 2.
Data Sharing Statement
