# Supplementary material for: Federally Qualified Health Centers and Performance of Medicare Accountable Care Organizations
Source: JAMA Netw Open. 2024 Nov 18;7(11):e2445536. doi: 10.1001/jamanetworkopen.2024.45536 (PMC11574694; doi:10.1001/jamanetworkopen.2024.45536)
Supplement: Supplement 1. — eMethods. Empirical Approaches eReferences. eTable 1. Sample Inclusion and Exclusion Criteria for the Difference-in-Difference Analysis eTable 2. Timing of ACOs Including FQHCs for the First Time, 2017-2022 eTable 3. Baseline Characteristics of MSSP ACOs in the Analytic Sample for the Difference-in-Difference Analysis eTable 4. Estimated Change in MSSP ACO-Assigned Beneficiaries, Utilization, Expenditure, and Quality Measures Associated With Participation of Federally Qualified Health Centers eTable 5. Comparison of Unadjusted p-values and Sharpened False Discovery Rate q-values for Multiple Comparison Adjustment eTable 6. Sensitivity Analysis: Estimated Percent Change in MSSP ACO-Assigned Beneficiaries, Utilization, and Expenditure Associated With Participation of Federally Qualified Health Centers using Logged Dependent Variables eTable 7. Sensitivity Analysis: Estimated Change in MSSP ACO-Assigned Beneficiaries, Utilization, Expenditure, and Quality Measures Associated With Participation of Federally Qualified Health Centers Using Alternative Specifications eTable 8. Sensitivity Analysis: Estimated Percentage-Point Change in MSSP ACO-Assigned Beneficiaries Associated With Participation of Federally Qualified Health Centers eTable 9. Sensitivity Analysis: Estimated Change in MSSP ACO Provider Compositions Associated With Participation of Federally Qualified Health Centers eFigure. Event Study Plots of Difference-in-Difference Analyses for Trends in Outcomes Among Treated and Control ACOs [file jamanetwopen-e2445536-s001.pdf]

Supplementary Online Content

Kun L, Hou Y, McStay F, Gonzalez-Smith J, Saunders RS. Federally qualified health centers and performance of Medicare accountable care organizations. *JAMA Netw Open*. 2024;7(11):e2445536. doi:10.1001/jamanetworkopen.2024.45536

eMethods. Empirical Approaches

eReferences.

eTable 1. Sample Inclusion and Exclusion Criteria for the Difference-in-Difference Analysis

eTable 2. Timing of ACOs Including FQHCs for the First Time, 2017-2022

eTable 3. Baseline Characteristics of MSSP ACOs in the Analytic Sample for the Difference-in-Difference Analysis

eTable 4. Estimated Change in MSSP ACO-Assigned Beneficiaries, Utilization, Expenditure, and Quality Measures Associated With Participation of Federally Qualified Health Centers

eTable 5. Comparison of Unadjusted p-values and Sharpened False Discovery Rate q-values for Multiple Comparison Adjustment

eTable 6. Sensitivity Analysis: Estimated Percent Change in MSSP ACO-Assigned Beneficiaries, Utilization, and Expenditure Associated With Participation of Federally Qualified Health Centers using Logged Dependent Variables

eTable 7. Sensitivity Analysis: Estimated Change in MSSP ACO-Assigned Beneficiaries, Utilization, Expenditure, and Quality Measures Associated With Participation of Federally Qualified Health Centers Using Alternative Specifications

eTable 8. Sensitivity Analysis: Estimated Percentage-Point Change in MSSP ACO-Assigned Beneficiaries Associated With Participation of Federally Qualified Health Centers

eTable 9. Sensitivity Analysis: Estimated Change in MSSP ACO Provider Compositions Associated With Participation of Federally Qualified Health Centers

eFigure. Event Study Plots of Difference-in-Difference Analyses for Trends in Outcomes Among Treated and Control ACOs

This supplementary material has been provided by the authors to give readers additional information about their work.

© 2024 Kun L et al. *JAMA Network Open.*

eMethods. Empirical Approaches

For each ACO-year-level outcome, we estimated the following difference-in-differences (DD) event-study specification:

$$Y_{it} = \beta_0 + Treated_i * [\sum_{j=-3}^{-2} \boldsymbol{\beta_j}\, \mathbf{1}\{t - t_k^* = j\} + \sum_{j=0}^{4} \boldsymbol{\delta_j} \mathbf{1}\{t - t_k^* = j\}] + ACO_i + Year_t + \varepsilon_{it},$$

where Y_{it} is the outcome of interest (beneficiary characteristics, utilization, spending, quality of care) for ACO i in calendar year t. $Treated_i = 1$ if the index ACO ever included FQHCs. t_k^* is the year when a treated ACO included first FQHCs. $\mathbf{1}\{t - t_k^* = j\} = 1$ when $t - t_k^* = j$. The ACO fixed effects (ACO_i) accounted for time-invariant differences before the inclusion of FQHCs between treated ACOs that included FQHCs and control ACOs that did not. The year-fixed effects ($Year_t$) adjusted for year trends. We did not include other time-varying covariates that can be potentially affected by FQHC inclusion. δ_j are the coefficient of interest, capturing the average differences in outcomes between treated ACOs and control ACOs, relative to the event time period -1 (where $j = -1$). Standard errors were clustered at the ACO level.

Callaway and Sant'Anna Approach. We used the Callaway and Sant'Anna approach to account for the staggered inclusion of FQHCs. We followed the recently recommended approach to correct the asymmetric construction of pre- and post-treatment coefficients under the Callaway and Sant'Anna approach.[1] Specifically, we estimated staggered difference-in-difference regressions using the Stata command *xthdidregress* with the recommended post-estimation command *eventbaseline* to generate ATET estimates.[2] We included estimates for the event time period from -3 to 4 to calculate the aggregated estimates.

Test for Pre-trends. We assessed the validity of the parallel trend assumption by testing the presence of differential pre-trends using a joint F-test. We tested and reported whether the estimated coefficients for up to 3 years before the FQHC inclusion (β_j) are equal and not statistically significant from zero. Our null hypothesis is $\beta_{-3} = \beta_{-2} = 0$. A significant test result ($p<0.05$) suggests evidence of pre-trend.

eReferences

1. Roth J. Interpreting Event-Studies from Recent Difference-in-Differences Methods. January 22, 2024. Accessed April 8, 2024. https://www.jonathandroth.com/assets/files/HetEventStudies.pdf

© 2024 Kun L et al. *JAMA Network Open.*

2. Koren M. EVENTBASELINE: Correct Event Study After XTHDIDREGRESS. [software]. Published online 2024. https://github.com/codedthinking/eventbaseline

© 2024 Kun L et al. *JAMA Network Open.*

eTable 1. Sample inclusion and exclusion criteria for the difference-in-difference analysis

Identification of sample	Number of ACOs	Number of ACO-years	Treated ACOs	Control ACOs
MSSP ACOs during 2016-2022	836	3,463	224	612
Exclude if observed data for only one year	693	3,320	196	497
Exclude if no pre-FQHC participation data (always treated)	581	2,803	84	497
Exclude if ACOs dropped all FQHCs in later years (switcher)	540	2,565	43	497

eTable 2. Timing of ACOs including FQHCs for the first time, 2017-2022

	Number of ACOs	Number of ACO-years
**First-time FQHC inclusion**		
2017	4	28
2018	6	28
2019	6	29
2020	7	37
2021	12	65
2022	8	36
**Never had FQHC participation**	497	2,342

© 2024 Kun L et al. *JAMA Network Open.*

eTable 3. Baseline Characteristics of MSSP ACOs in the analytic sample for the difference-in-difference analysis[a]

Outcomes	ACOs that never had FQHC participation, Mean (SD)	ACOs that included FQHCs during the study period, Mean (SD)	p-value[b]
Number of ACOs	497	43	
ACO-assigned beneficiaries			
Total person-years	16061.9 (13677.5)	17023.6 (15314.7)	0.662
Dual person-years	1067.9 (1035.4)	1114.5 (1193.5)	0.780
Disabled person-years	1801 (1607.7)	2212.1 (1915.7)	0.114
Racial and ethnic minoritized person-years[c]	2353.5 (2473.3)	2355.6 (2539.9)	0.996
Utilization[d]			
FQHC and RHC visits	223.5 (667.5)	269 (625.5)	0.666
Total primary care visits	10626.2 (2353.6)	10410.7 (1502.2)	0.555
Outpatient emergency department visits	709.4 (189.3)	718.2 (156.9)	0.769
Short-term acute care hospital discharges	297.1 (100.3)	290.1 (48.6)	0.651
Skilled nursing facility discharges	71.8 (73.7)	62 (24.2)	0.387
Per capita expenditure, $	11086.9 (3386.4)	10415.7 (1679.1)	0.199
Quality[e], %			
Influenza immunization	70.1 (13)	67.4 (12)	0.183
Tobacco use screening and cessation intervention	85.2 (16.1)	79.2 (18)	0.022
Screening for depression and follow-up plan	56.5 (21.9)	55.3 (21.7)	0.724
Colorectal cancer screening	63.3 (14.2)	62.9 (11.5)	0.855
Breast cancer screening	68.8 (12.6)	69.8 (9.0)	0.602
Depression remission at 12 months	7.8 (12.9)	8.3 (10.9)	0.811
Diabetes control: (HbA1c <=9%)	82.6 (10.6)	81.9 (6.5)	0.665
Controlling high blood pressure	71.3 (8.8)	70.1 (7.0)	0.397

Abbreviations: MSSP, Medicare Shared Saving Program; ACO, accountable care organization; FQHC, federally qualified health centers; RHC, rural health clinic; ED, emergency department; HbA1c, hemoglobin A1c.

[a] Estimated in the year when an ACO first appeared in the data. [b] Two-sided t-test. [c] Includes all beneficiaries who are American Indian or Alaska Native, Asian and Pacific Islander, Black, Hispanic, Other, and Unknown. [d] Measures are presented in per 1,000 person-years. [e] Measures are presented as percentage of patients. [e] Advanced practitioners include physician assistants, registered nurses, and certified nurse midwives.

© 2024 Kun L et al. *JAMA Network Open.*

eTable 4. Estimated Change in MSSP ACO-Assigned Beneficiaries, Utilization, Expenditure, and Quality Measures Associated with Participation of Federally Qualified Health Centers

Outcomes	Total person-year	Dual person-year	Disabled person-year	Minoritized person-year	FQHC & RHC visits	Primary care visits	ED visits	Hospital discharges	SNF discharges
	Coefficient (SE)	Coefficient (SE)	Coefficient (SE)	Coefficient (SE)	Coefficient (SE)	Coefficient (SE)	Coefficient (SE)	Coefficient (SE)	Coefficient (SE)
Time Period									
-3	-291.4	-101.8	-74.2	-18.0	4.1	-149.1	-15.9	-9.9	-0.1
	(1,538.4)	(104.3)	(217.8)	(175.4)	(63.2)	(173.3)	(21.6)	(8.2)	(3.7)
-2	425.7	23.2	52.1	61.6	-4.1	4.9	-0.6	-2.3	0.0
	(728.5)	(63.7)	(87.3)	(140.4)	(43.2)	(105.0)	(14.0)	(5.5)	(2.5)
-1	Reference	Reference	Reference	Reference	Reference	Reference	Reference	Reference	Reference
	-	-	-	-	-	-	-	-	-
0	11,507.0**	1,073.3**	1,567.0**	1,562.8**	617.3**	-91.7	33.9*	4.9	1.4
	(3,880.7)	(323.6)	(446.5)	(464.1)	(191.8)	(96.0)	(13.5)	(3.9)	(2.3)
1	13,879.1**	1,383.6**	1,763.4**	2,131.6**	740.6*	-136.0	28.4	2.5	0.1
	(5,062.9)	(451.5)	(539.5)	(615.7)	(294.7)	(197.2)	(18.0)	(6.2)	(3.6)
2	17,928.6**	1,458.1*	1,966.0*	2,311.0*	366.7**	278.5	7.0	4.8	4.8
	(8,765.4)	(597.7)	(830.7)	(1,013.8)	(98.6)	(253.9)	(25.3)	(7.0)	(4.3)
3	2,449.6	265.7*	325.6	470.5	363.3**	530.6	-20.9	6.8	7.3*
	(3,798.9)	(126.4)	(305.5)	(561.5)	(114.9)	(320.9)	(34.5)	(9.1)	(3.3)
4	2,429.8	52.7	29.4	350.7	384.8**	807.4	-42.3	12.8	11.2*
	(2,115.2)	(88.2)	(184.6)	(334.6)	(127.1)	(506.1)	(46.3)	(11.0)	(5.1)
Observations[a]	2,565	2,565	2,565	2,565	2,565	2,565	2,565	2,565	2,565
Outcomes	Per capita expenditure	Influenza immunization	Tobacco screening	Depression screening	Colorectal cancer screening	Breast cancer screening	Depression remission	Diabetes control	Blood pressure control
	Coefficient (SE)	Coefficient (SE)	Coefficient (SE)	Coefficient (SE)	Coefficient (SE)	Coefficient (SE)	Coefficient (SE)	Coefficient (SE)	Coefficient (SE)
Time period									
-3	-210.2	1.2	-3.4	1.2	1.4	2.1	0.6	2.0*	0.0
	(238.9)	(2.2)	(4.5)	(3.6)	(2.2)	(1.9)	(4.1)	(1.0)	(1.0)
-2	-55.8	-1.0	-1.9	-0.3	0.3	1.0	-0.9	0.3	0.7
	(184.5)	(1.4)	(3.4)	(1.9)	(1.7)	(1.2)	(3.4)	(0.7)	(0.8)
-1	Reference	Reference	Reference	Reference	Reference	Reference	Reference	Reference	Reference
	-	-	-	-	-	-	-	-	-
0	32.5	1.4	7.9**	3.0	-0.2	-0.6	-0.7	0.1	0.1
	(133.0)	(1.7)	(2.8)	(2.3)	(1.6)	(1.2)	(1.8)	(1.4)	(0.9)
1	-99.9	1.9	5.4	5.5	-1.6	-2.5	0.6	1.8	0.5
	(191.2)	(2.1)	(4.2)	(3.1)	(2.0)	(1.9)	(3.0)	(1.2)	(1.1)
2	166.5	6.0	15.3**	6.9	3.5	1.6	6.0	-0.3	-0.1
	(145.2)	(3.5)	(5.1)	(4.9)	(2.4)	(1.7)	(4.4)	(3.3)	(1.4)
3	89.1	8.4**	11.7	13.1*	3.2	0.4	2.7	3.0	2.1
	(162.2)	(3.0)	(7.0)	(5.6)	(2.7)	(1.8)	(5.3)	(2.6)	(1.3)
4	213.2	12.2**	10.0*	17.8*	4.8*	2.2	6.8	4.1	2.5
	(242.3)	(4.2)	(4.8)	(7.5)	(2.2)	(2.4)	(7.3)	(3.4)	(2.3)
Observations	2,565	2,532	2,531	2,545	2,530	2,530	2,479	2,545	2,542

Abbreviations: MSSP, Medicare Shared Saving Program; ACO, accountable care organization; FQHC, federally qualified health centers; RHC, rural health clinic; ED, emergency department; SNF, skilled nursing facility.

[a]Analyses were limited to ACO-years with non-missing data for each outcome; the corresponding observations for each outcome are reported.

** p<0.01, * p<0.05.

© 2024 Kun L et al. *JAMA Network Open.*

eTable 5. Comparison of Unadjusted p-values and Sharpened False Discovery Rate q-values for Multiple Comparison Adjustment

Outcomes	Estimated change	Unadjusted p-value	Sharpened q-value
Assigned beneficiaries			
Total person-years	9594.1	0.01	0.02
Dual person-years	872.9	<.001	0.01
Disabled person-years	1137.6	<.001	0.01
Racial and ethnic minoritized person-years[b]	1350.8	<.001	0.01
Utilization[c]			
FQHC and RHC visits	494.5	<.001	<.001
Total primary care visits	325.8	0.17	0.19
Outpatient emergency department visits	6.7	0.79	0.45
Short-term acute care hospital discharges	10.4	0.15	0.17
Skilled nursing facility discharges	5.0	0.08	0.10
Per capita expenditure, $	169.0	0.31	0.30
Quality[d], %			
Influenza immunization	5.9	0.01	0.02
Tobacco use screening and cessation intervention	11.8	0.00	0.02
Screening for depression and follow-up plan	8.9	0.04	0.06
Colorectal cancer screening	1.3	0.48	0.38
Breast cancer screening	-0.8	0.57	0.40
Depression remission at 12 months	3.2	0.42	0.35
Diabetes control: (HbA1c <=9%)	0.9	0.62	0.40
Controlling high blood pressure	0.8	0.52	0.38

Abbreviations: MSSP, Medicare Shared Saving Program; ACO, accountable care organization; FQHC, federally qualified health centers; RHC, rural health clinic; ED, emergency department.

[a] Means of outcomes across all pre-treatment years among ACOs which included federally qualified health center during 2016-2022. [b] Includes all beneficiaries who are American Indian or Alaska Native, Asian and Pacific Islander, Black, Hispanic, Other, and Unknown. [c] Measures are presented in per 1,000 person-years. [d] Measures are presented as percentage of patients.

© 2024 Kun L et al. *JAMA Network Open.*

eTable 6. Sensitivity Analysis: Estimated Percent Change in MSSP ACO-Assigned Beneficiaries, Utilization, and Expenditure Associated with Participation of Federally Qualified Health Centers using Logged Dependent Variables

Outcomes	Total person-year	Dual person-year	Disabled person-year	Minoritized person-year	FQHC & RHC visits	Primary care visits	ED visits	Short-term hospital discharges	SNF discharges	Per capita expenditure
	Estimated percent change (SE)	Estimated percent change (SE)	Estimated percent change (SE)	Estimated percent change (SE)	Estimated percent change (SE)	Estimated percent change (SE)	Estimated percent change (SE)	Estimated percent change (SE)	Estimated percent change (SE)	Estimated percent change (SE)
ATET[a]	48.8**	70.2**	67.7**	57.0**	490.9**	3.8	2.7	3.5	3.8	1.3
	(0.1)	(0.1)	(0.1)	(0.1)	(0.3)	(0.0)	(0.0)	(0.0)	(0.1)	(0.0)
Time period										
-3	-1.5	-15.2	-7.6	-5.0	-5.3	-1.7	-3.7	-3.9	-0.0	-1.5
	(0.1)	(0.2)	(0.1)	(0.1)	(0.3)	(0.0)	(0.0)	(0.0)	(0.1)	(0.0)
-2	2.2	2.4	5.0	1.5	10.3	-0.3	-0.4	-0.6	2.0	-0.0
	(0.1)	(0.1)	(0.1)	(0.1)	(0.2)	(0.0)	(0.0)	(0.0)	(0.1)	(0.0)
-1	Reference	Reference	Reference	Reference	Reference	Reference	Reference	Reference	Reference	Reference
	-	-	-	-	-	-	-	-	-	-
0	48.1**	83.1**	80.7**	55.8**	528.2**	-0.5	5.2*	1.7	3.7	0.7
	(0.1)	(0.1)	(0.1)	(0.1)	(0.3)	(0.0)	(0.0)	(0.0)	(0.0)	(0.0)
1	68.4**	114.3**	106.2**	82.5**	775.3**	-0.7	4.8	0.8	0.5	-0.4
	(0.1)	(0.2)	(0.1)	(0.2)	(0.3)	(0.0)	(0.0)	(0.0)	(0.1)	(0.0)
2	90.0**	107.4**	105.2**	91.7**	587.0**	3.1	2.9	2.2	7.2	1.5
	(0.2)	(0.3)	(0.2)	(0.3)	(0.4)	(0.0)	(0.0)	(0.0)	(0.1)	(0.0)
3	28.4	18.7	36.5*	30.2	459.3**	5.5	-1.6	2.2	4.4	0.3
	(0.2)	(0.1)	(0.2)	(0.2)	(0.3)	(0.0)	(0.0)	(0.0)	(0.1)	(0.0)
4	21.3	17.0*	20.9*	26.8**	267.0**	8.2	-4.5	3.0	6.7	1.6
	(0.1)	(0.1)	(0.1)	(0.1)	(0.4)	(0.0)	(0.0)	(0.0)	(0.1)	(0.0)
Observations[b]	2,565	2,527	2,562	2,565	2,552	2,565	2,565	2,565	2,565	2,565

[a] Estimated percent changes of outcomes aggregated across 4 years following the inclusion of first FQHCs.

[b] Observations with zero counts were excluded.

Abbreviations: ATET, average treatment effect on the treated; MSSP, Medicare Shared Saving Program; ACO, accountable care organization; FQHC, federally qualified health centers; RHC, rural health clinic; ED, emergency department; SNF, skilled nursing facility.

** p<0.01, * p<0.05.

© 2024 Kun L et al. *JAMA Network Open.*

eTable 7. Sensitivity Analysis: Estimated Change in MSSP ACO-Assigned Beneficiaries, Utilization, Expenditure, and Quality Measures Associated with Participation of Federally Qualified Health Centers Using Alternative Specifications

	Baseline two-way fixed effects model without adjusting for potential heterogeneous treatment effects due to staggered FQHC inclusion		Adding ACOs that included FQHCs and dropped all FQHCs in later years	
Outcomes	Estimated change	(95% CI)	Estimated change	(95% CI)
Assigned beneficiaries				
Total person-years	11331.4	(3556.3 to 19106.4)	9027.0	(2288.6 to 15765.5)
Dual person-years	1121.9	(443.1 to 1800.7)	797.8	(324.8 to 1270.8)
Disabled person-years	1460.9	(637.5 to 2284.4)	1091.9	(411.2 to 1772.5)
Racial and ethnic minoritized person-years[b]	1683.4	(635.9 to 2730.9)	1273.4	(433 to 2113.7)
Utilization[c]				
FQHC and RHC visits	700.5	(297.6 to 1103.4)	468.2	(254.8 to 681.5)
Total primary care visits	-42.7	(-270.7 to 185.2)	307.8	(-125.7 to 741.3)
Outpatient emergency department visits	32.5	(-1.8 to 66.8)	7.8	(-38.9 to 54.4)
Short-term acute care hospital discharges	8.0	(-1.6 to 17.6)	9.0	(-4.4 to 22.4)
Skilled nursing facility discharges	2.2	(-2.2 to 6.6)	5.8	(-0.01 to 11.5)
Per capita expenditure, $	86.0	(-175.8 to 347.9)	153.5	(-153.3 to 460.2)
Quality[d], %				
Influenza immunization	1.0	(-2.0 to 4.0)	4.7	(0.4 to 9)
Tobacco use screening and cessation intervention	5.9	(-0.1 to 11.8)	11.0	(3.6 to 18.5)
Screening for depression and follow-up plan	3.3	(-1.1 to 7.8)	7.7	(-0.6 to 16.1)
Colorectal cancer screening	-1.8	(-4.6 to 0.9)	0.8	(-2.7 to 4.4)
Breast cancer screening	-1.6	(-3.9 to 0.7)	-1.7	(-4.7 to 1.3)
Depression remission at 12 months	0.7	(-2.2 to 3.6)	2.9	(-4.1 to 9.8)
Diabetes control: (HbA1c <=9%)	-0.9	(-3.2 to 1.4)	0.9	(-2.6 to 4.4)
Controlling high blood pressure	-0.6	(-2.1 to 0.9)	0.4	(-2.0 to 2.8)

Abbreviations: MSSP, Medicare Shared Saving Program; ACO, accountable care organization; FQHC, federally qualified health centers; CI, confidence interval; RHC, rural health clinic; ED, emergency department.

** p<0.01, * p<0.05.

© 2024 Kun L et al. *JAMA Network Open.*

eTable 8. Sensitivity Analysis: Estimated Percentage-Point Change in MSSP ACO-Assigned Beneficiaries Associated with Participation of Federally Qualified Health Centers

Outcomes	Percentage of dual person-year	Percentage of disabled person-year	Percentage of minoritized person-year
	Estimated percent change (SE)	Estimated percent change (SE)	Estimated percent change (SE)
ATET[a]	1.26	0.61	0.49
	(0.71)	(0.65)	(0.92)
Time period			
-3	-0.92	-0.77	-0.58
	(0.76)	(0.76)	(1.28)
-2	0.06	0.30	-0.02
	(0.35)	(0.40)	(0.55)
-1	Reference	Reference	Reference
	-	-	-
0	1.73*	2.19**	0.45
	(0.77)	(0.63)	(0.98)
1	2.89	2.01*	1.38
	(1.71)	(0.82)	(1.51)
2	0.57	0.11	-0.46
	(0.59)	(0.68)	(1.44)
3	0.21	-0.40	0.22
	(0.53)	(0.72)	(0.61)
4	-0.54	-1.65	-0.16
	(0.58)	(0.99)	(1.02)
Observations	2,565	2,565	2,565

[a] Estimated percentage-point changes of outcomes aggregated across 4 years following the inclusion of first FQHCs.

Abbreviations: ATET, average treatment effect on the treated; MSSP, Medicare Shared Saving Program; ACO, accountable care organization.

** p<0.01, * p<0.05.

© 2024 Kun L et al. *JAMA Network Open.*

eTable 9. Sensitivity Analysis: Estimated Change in MSSP ACO Provider Compositions Associated with Participation of Federally Qualified Health Centers

Outcomes	Number of Rural Health Clinics	Number of Critical Access Hospitals
	Estimated change<br>(SE)	Estimated change<br>(SE)
ATET	3.0	0.6
	(1.6)	(0.7)
Time period		
-3	-0.0	-0.1
	(0.7)	(0.2)
-2	0.3	0.2*
	(0.3)	(0.1)
-1	Reference	Reference
	-	-
0	2.0	0.9
	(1.2)	(0.6)
1	2.3	1.2
	(1.7)	(1.0)
2	4.8	1.7
	(3.5)	(1.4)
3	2.8	0.4
	(1.7)	(1.0)
4	3.6	-0.7
	(2.7)	(0.5)
Observations	2,565	2,565

Abbreviations: ATET, average treatment effect on the treated; MSSP, Medicare Shared Saving Program; ACO, accountable care organization.

** $p<0.01$, * $p<0.05$.

© 2024 Kun L et al. *JAMA Network Open.*

eFigure. Event Study Plots of Difference-in-Difference Analyses for Trends in Outcomes Among Treated and Control ACOs

Total person-years

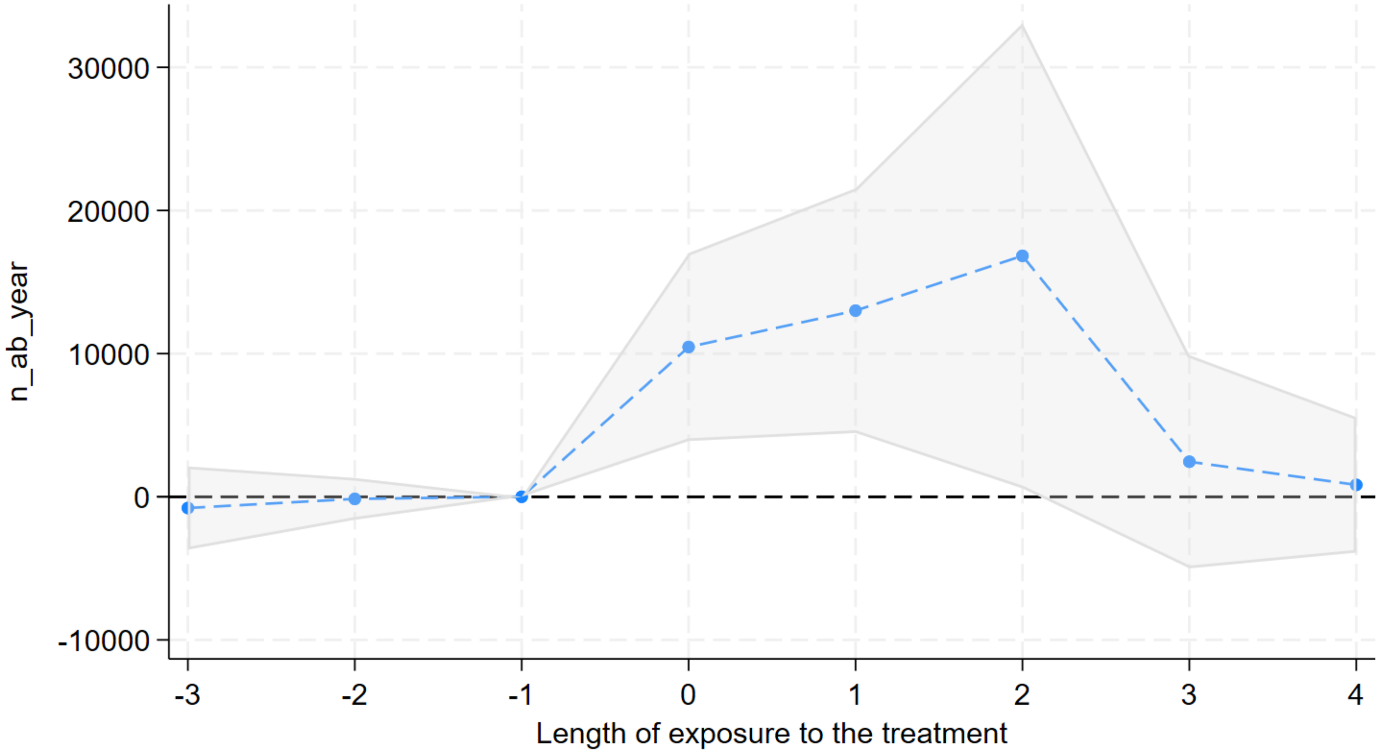

Dual person-years

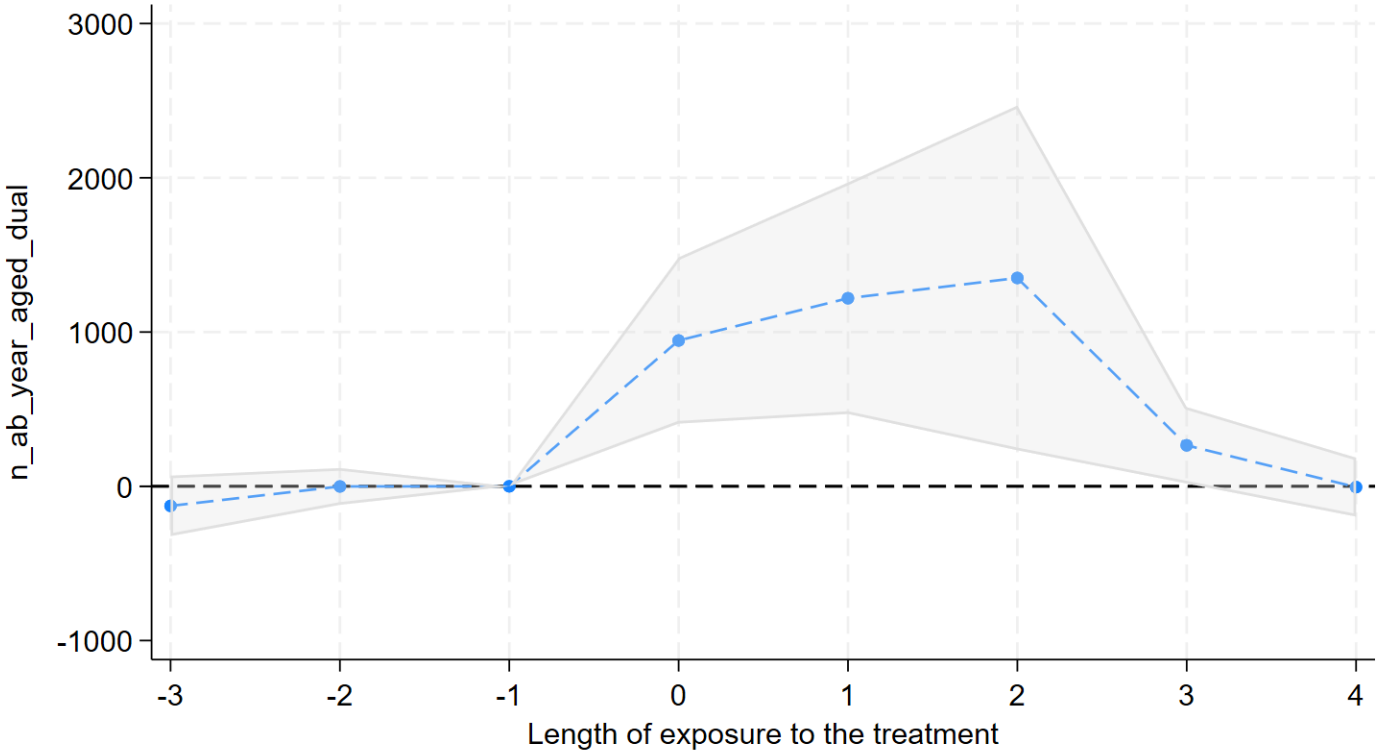

© 2024 Kun L et al. *JAMA Network Open.*

Disabled person-years

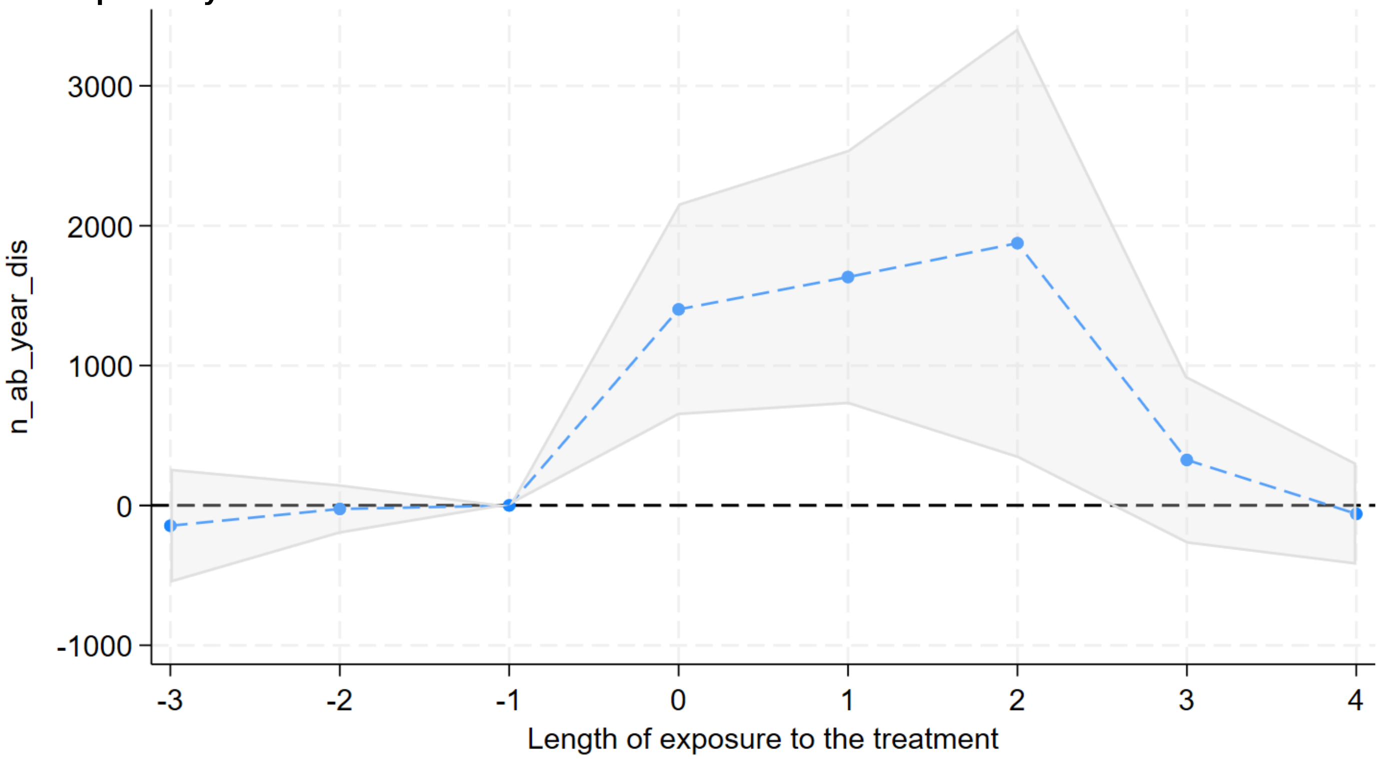

Racial and ethnic minoritized person-years

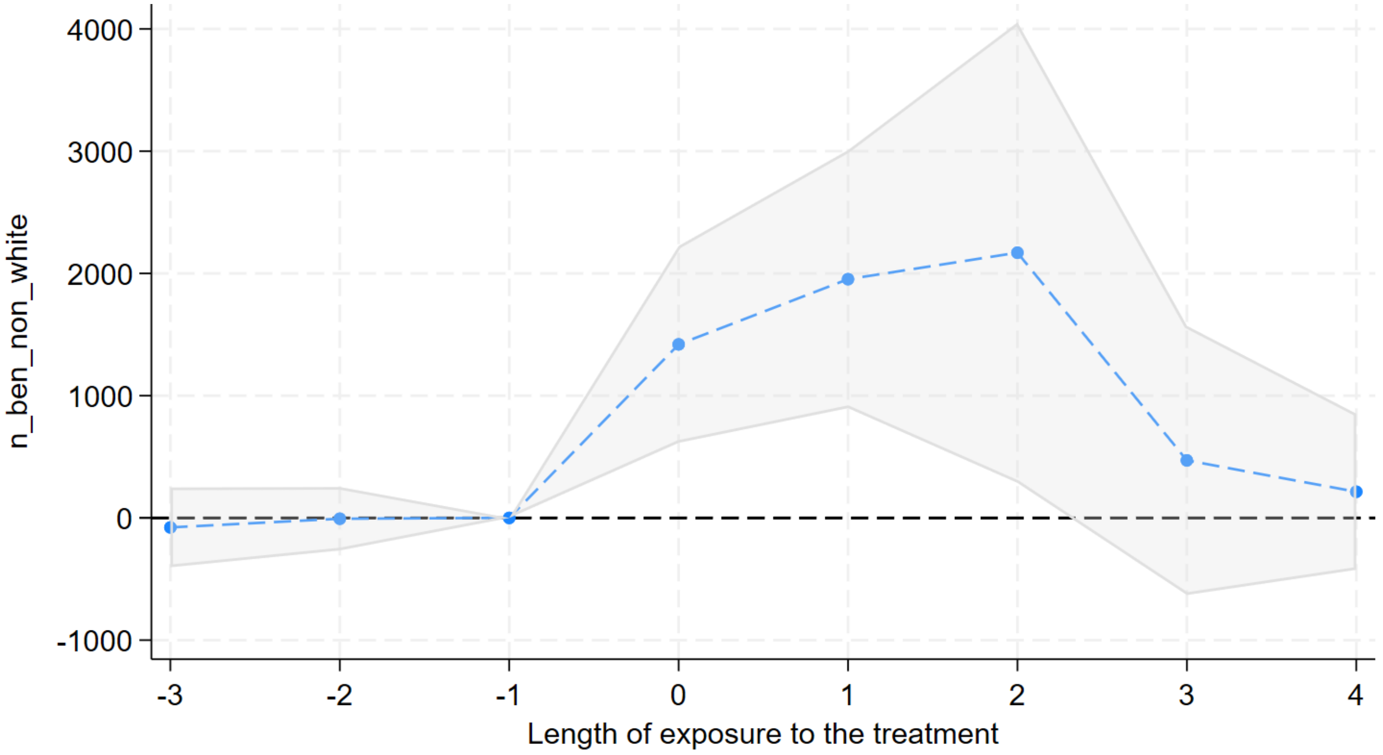

© 2024 Kun L et al. *JAMA Network Open.*

FQHC and RHC visits

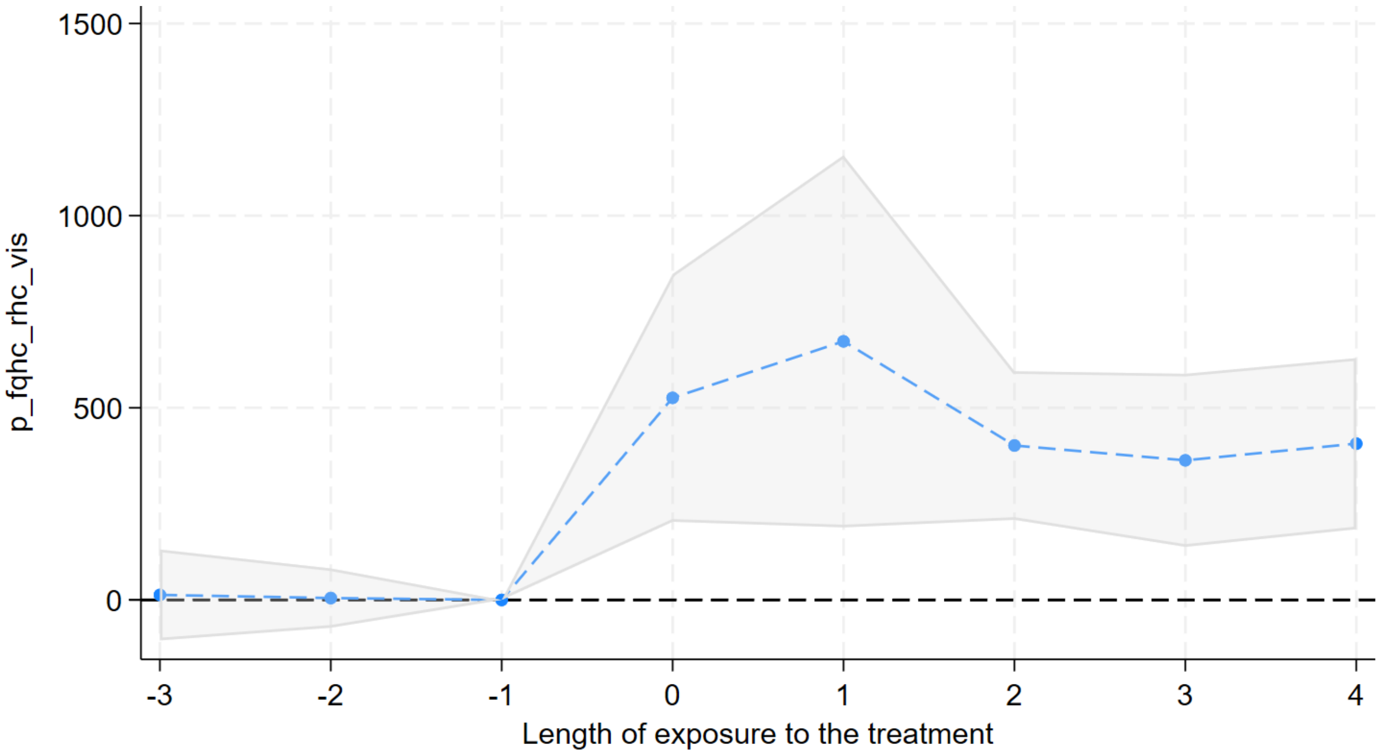

Total primary care visits

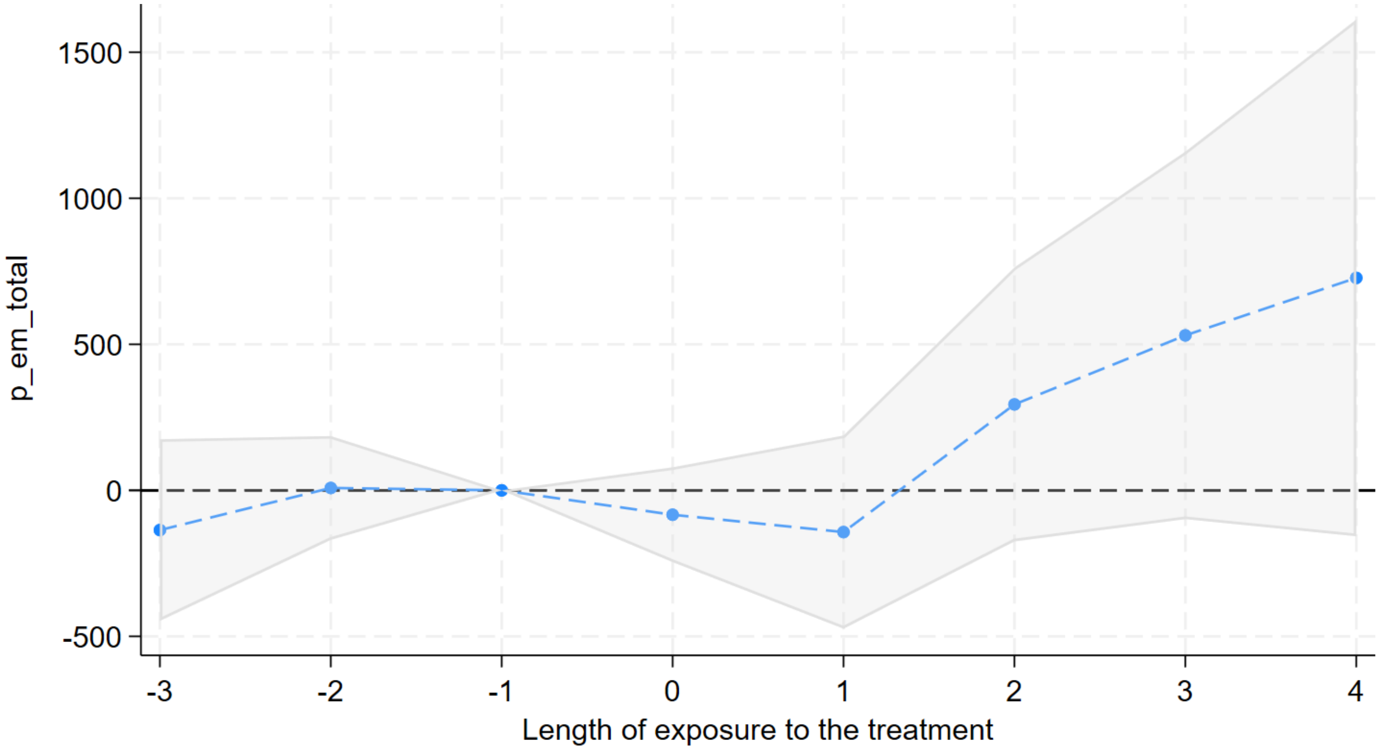

© 2024 Kun L et al. *JAMA Network Open.*

Outpatient emergency department visits

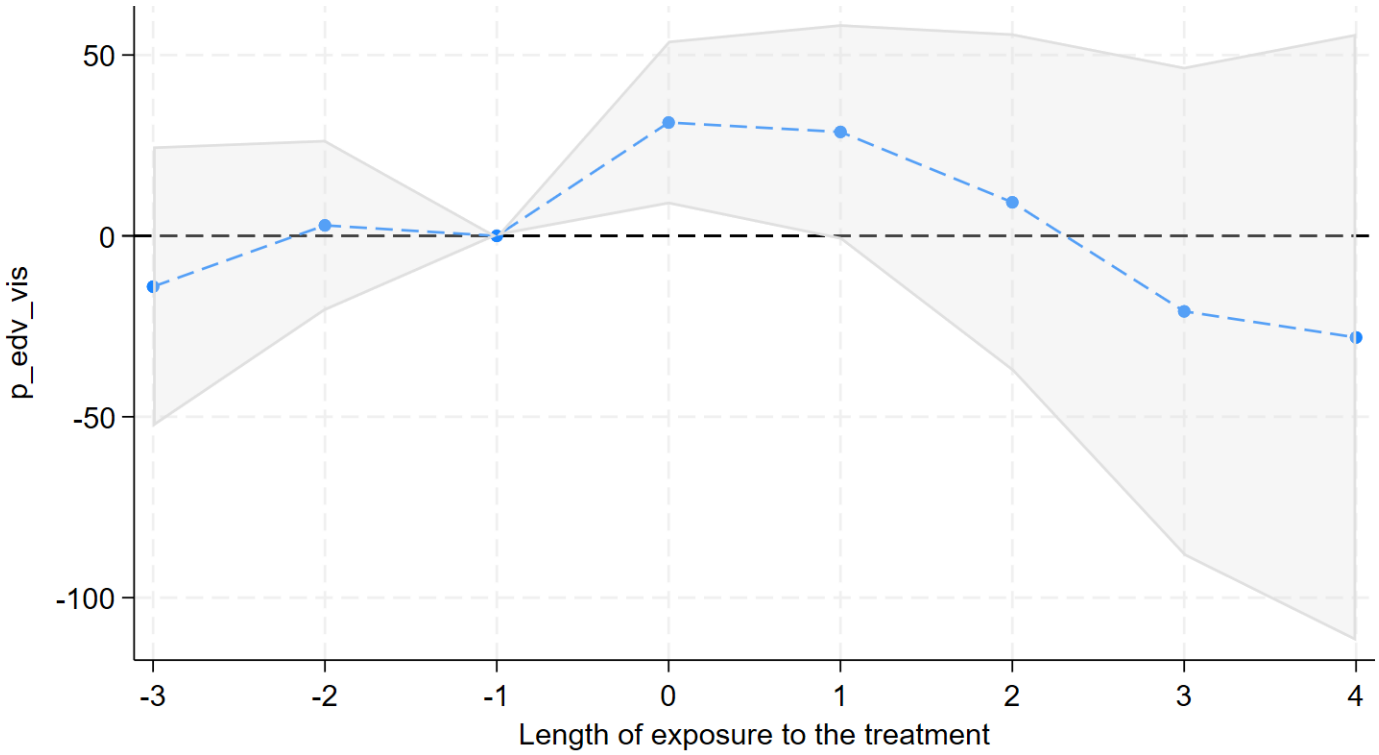

Short-term acute care hospital discharges

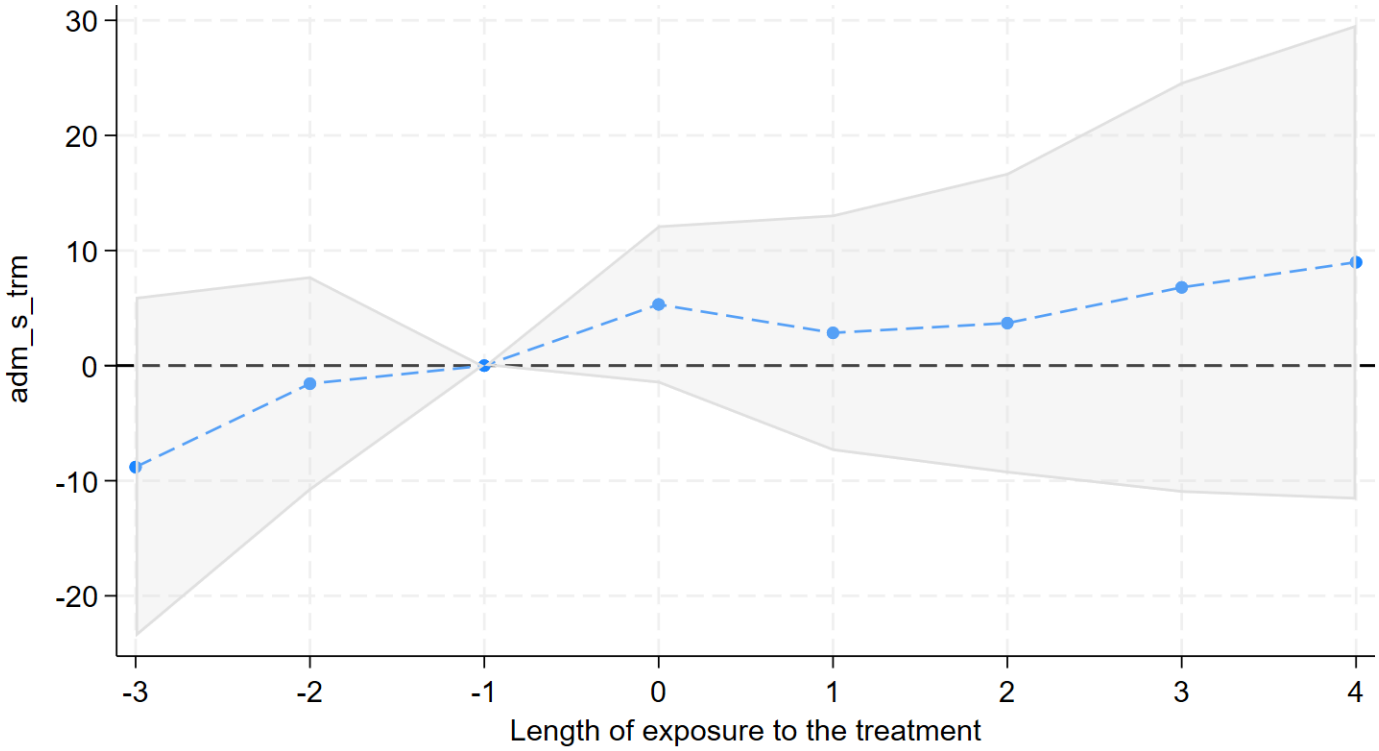

© 2024 Kun L et al. *JAMA Network Open.*

Skilled nursing facility discharges

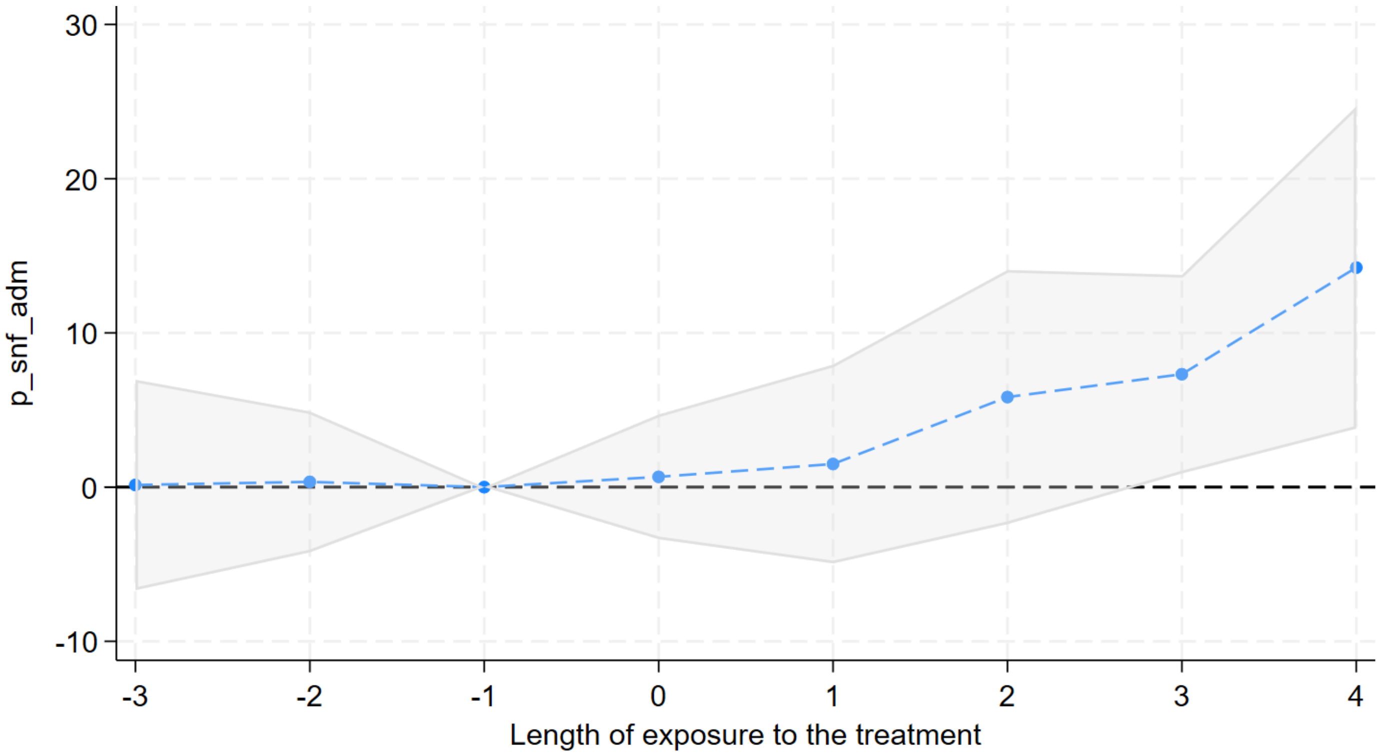

Per capita expenditure

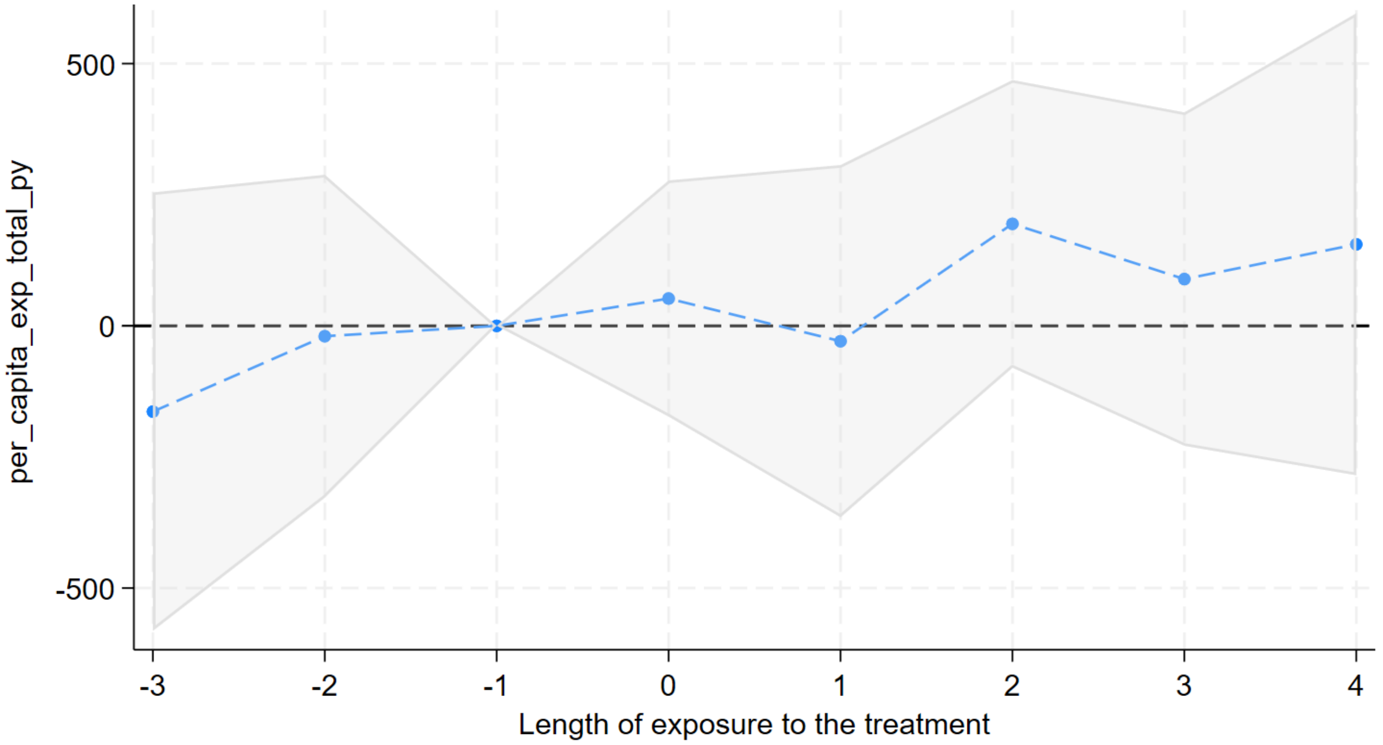

© 2024 Kun L et al. *JAMA Network Open.*

Influenza immunization

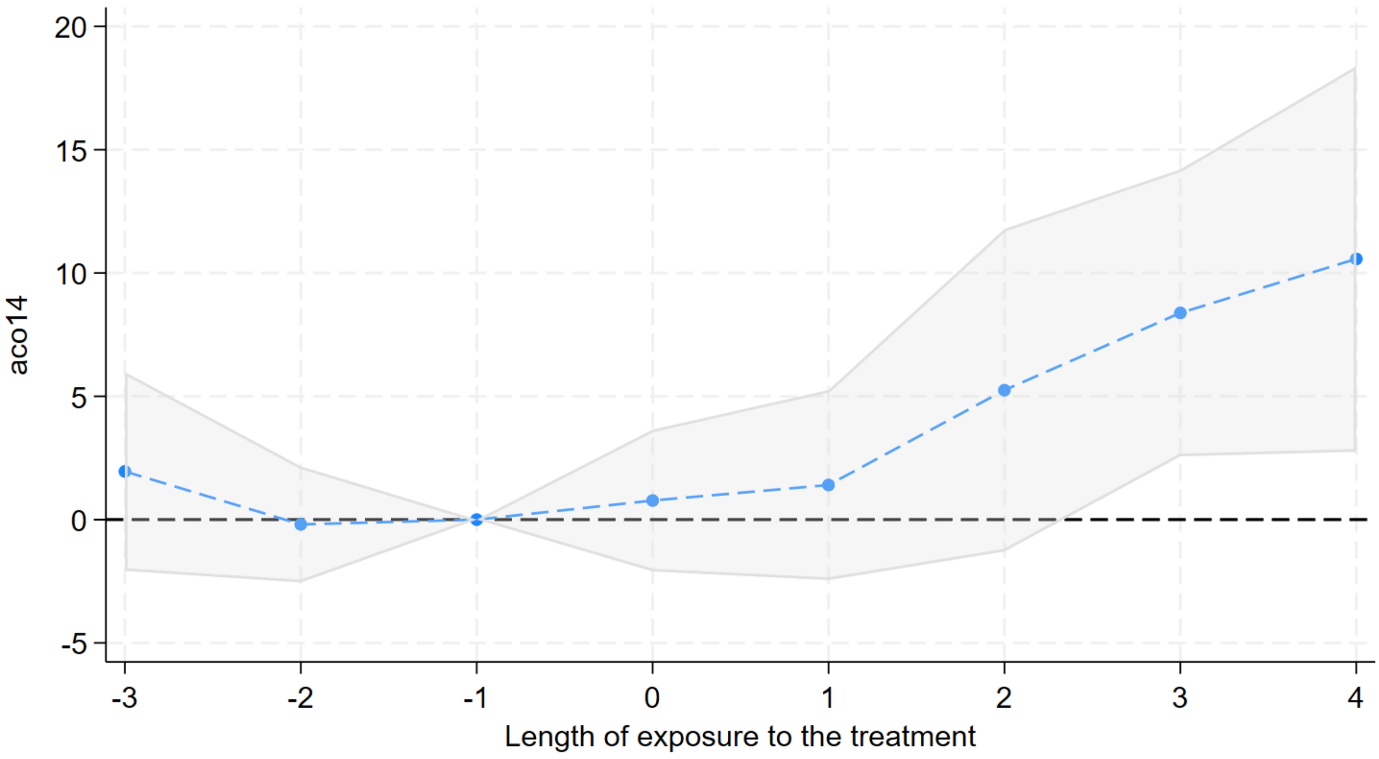

Tobacco use screening and cessation intervention

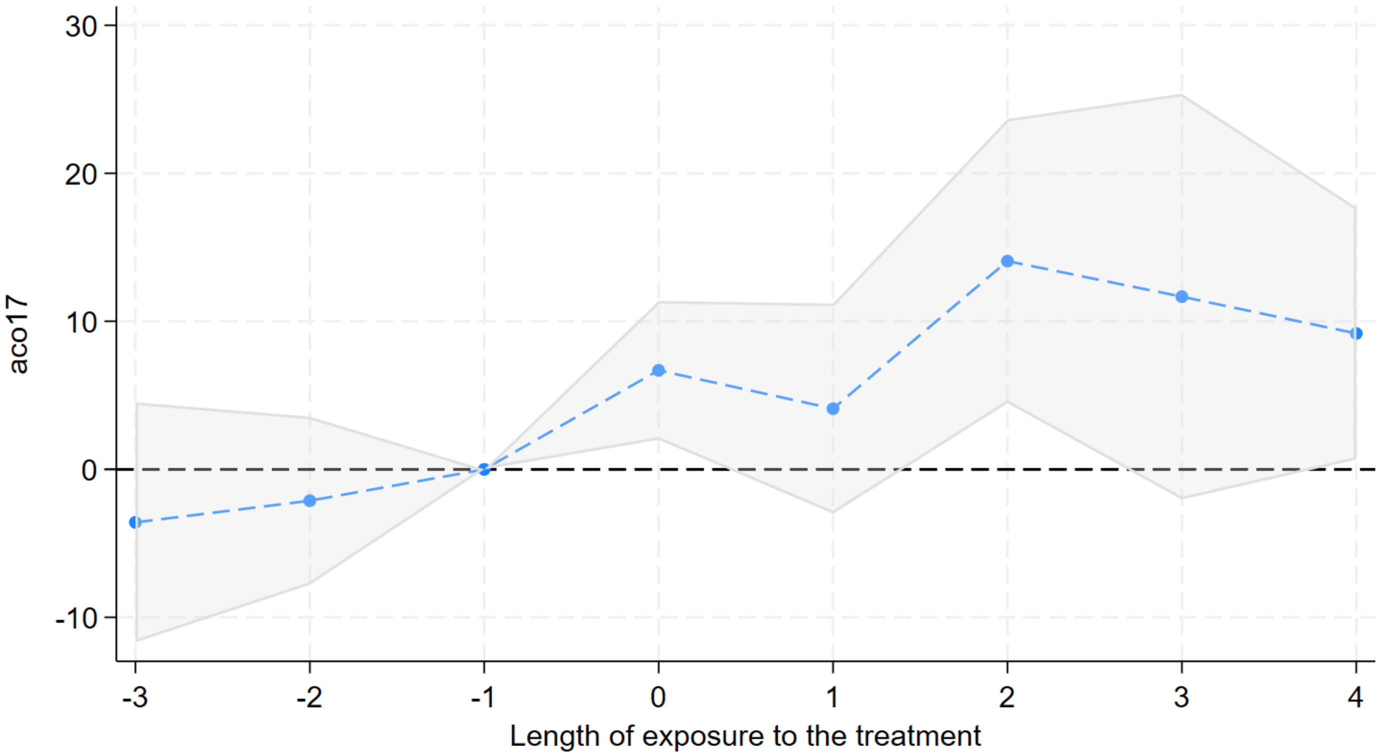

© 2024 Kun L et al. *JAMA Network Open.*

Screening for depression and follow-up plan

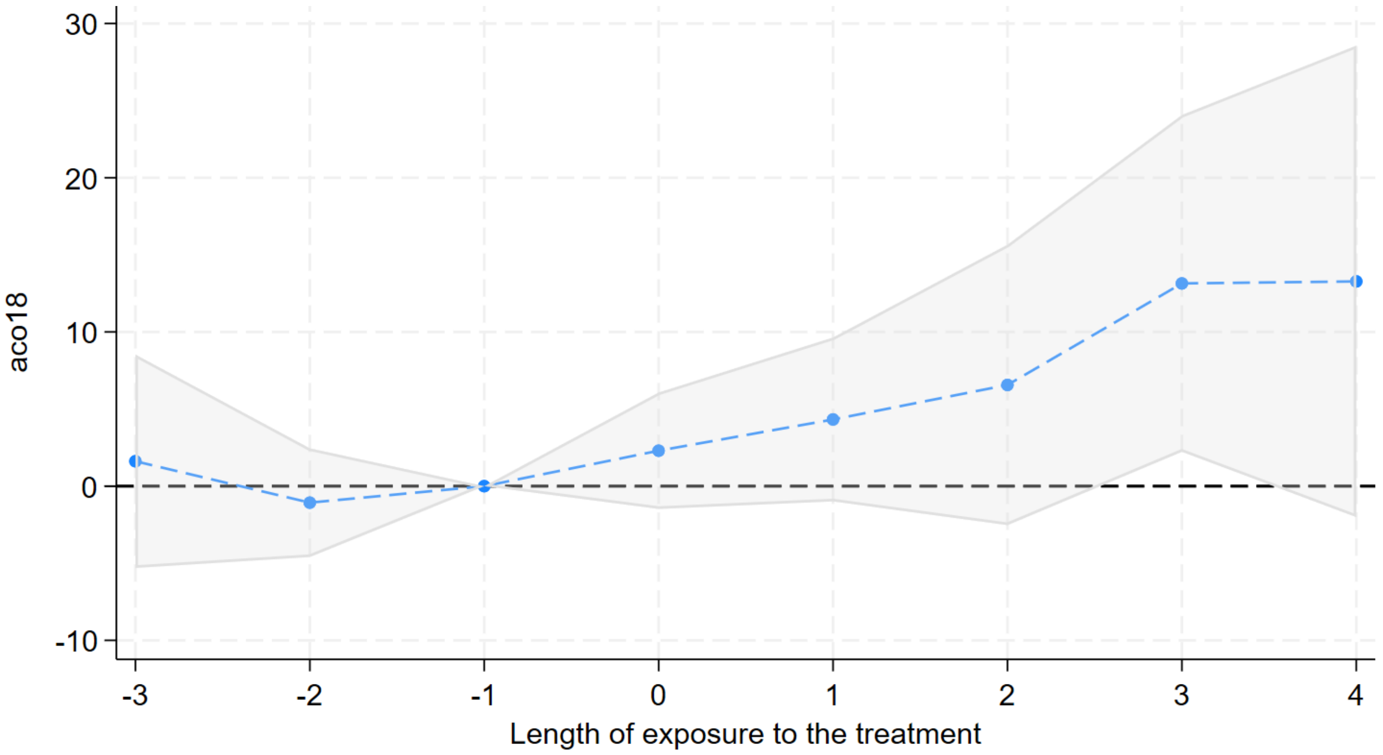

Colorectal cancer screening

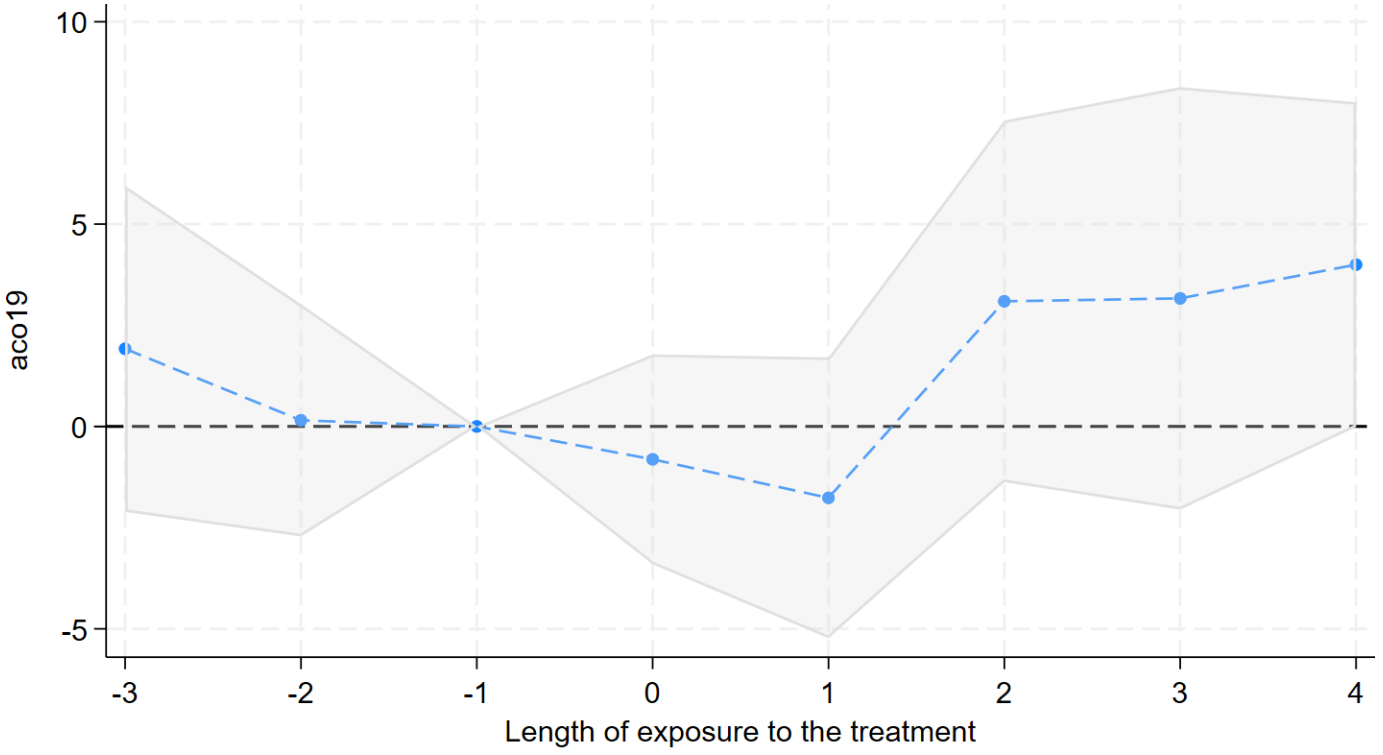

© 2024 Kun L et al. *JAMA Network Open.*

Breast cancer screening

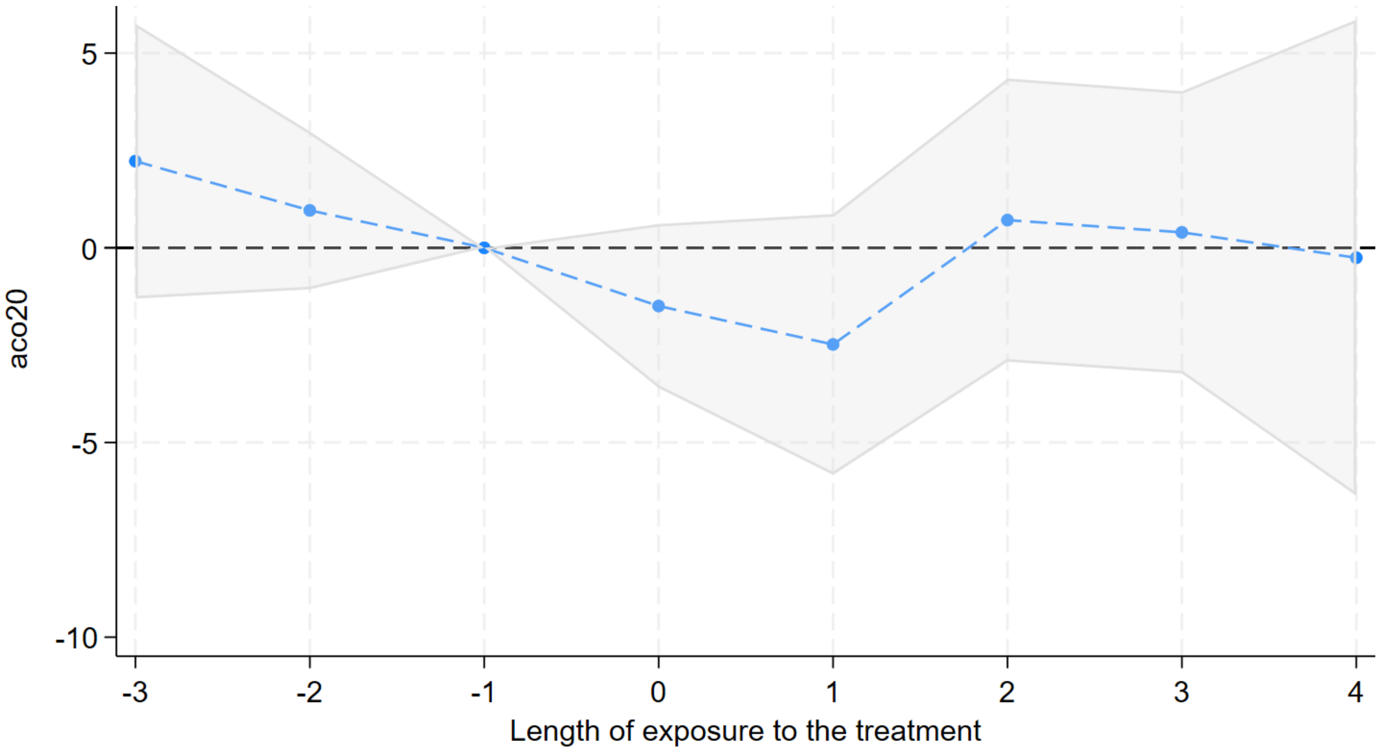

Depression remission at 12 months

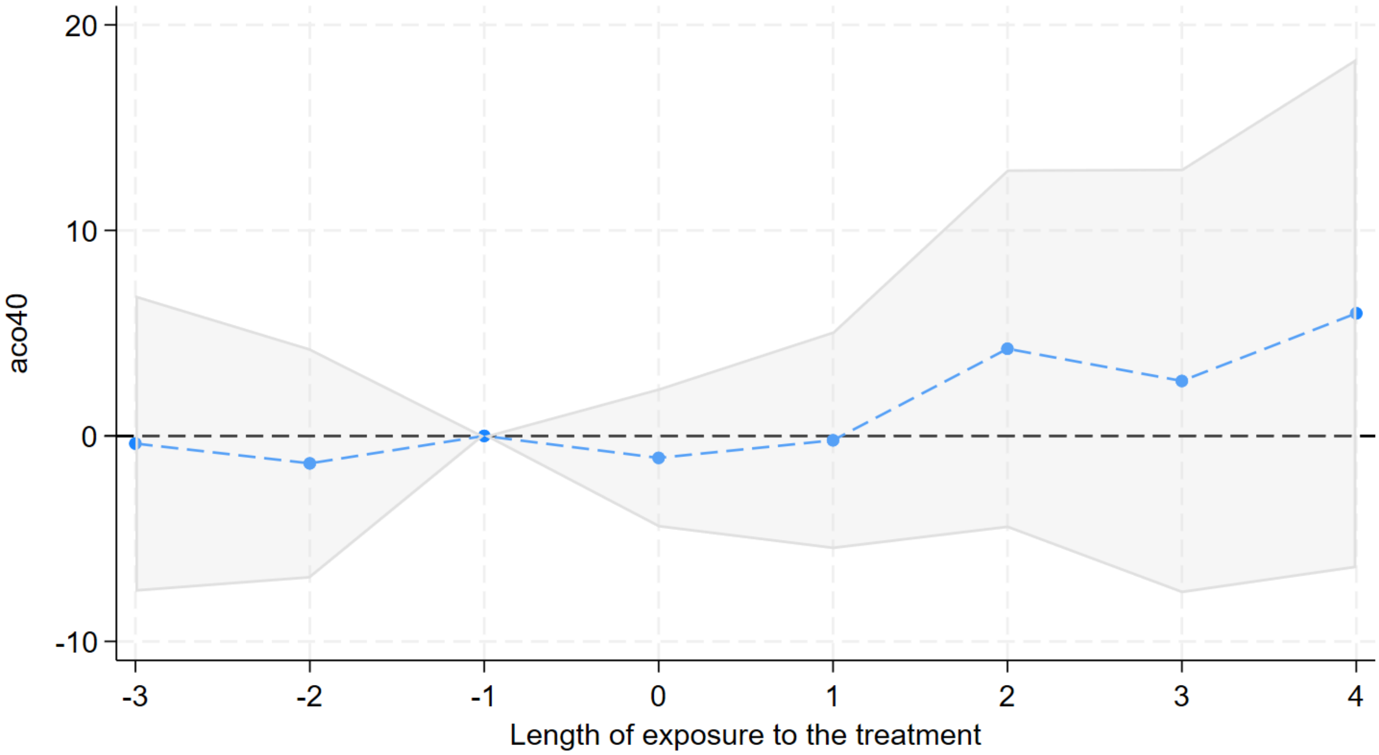

© 2024 Kun L et al. *JAMA Network Open.*

Diabetes control: (HbA1c <=9%)

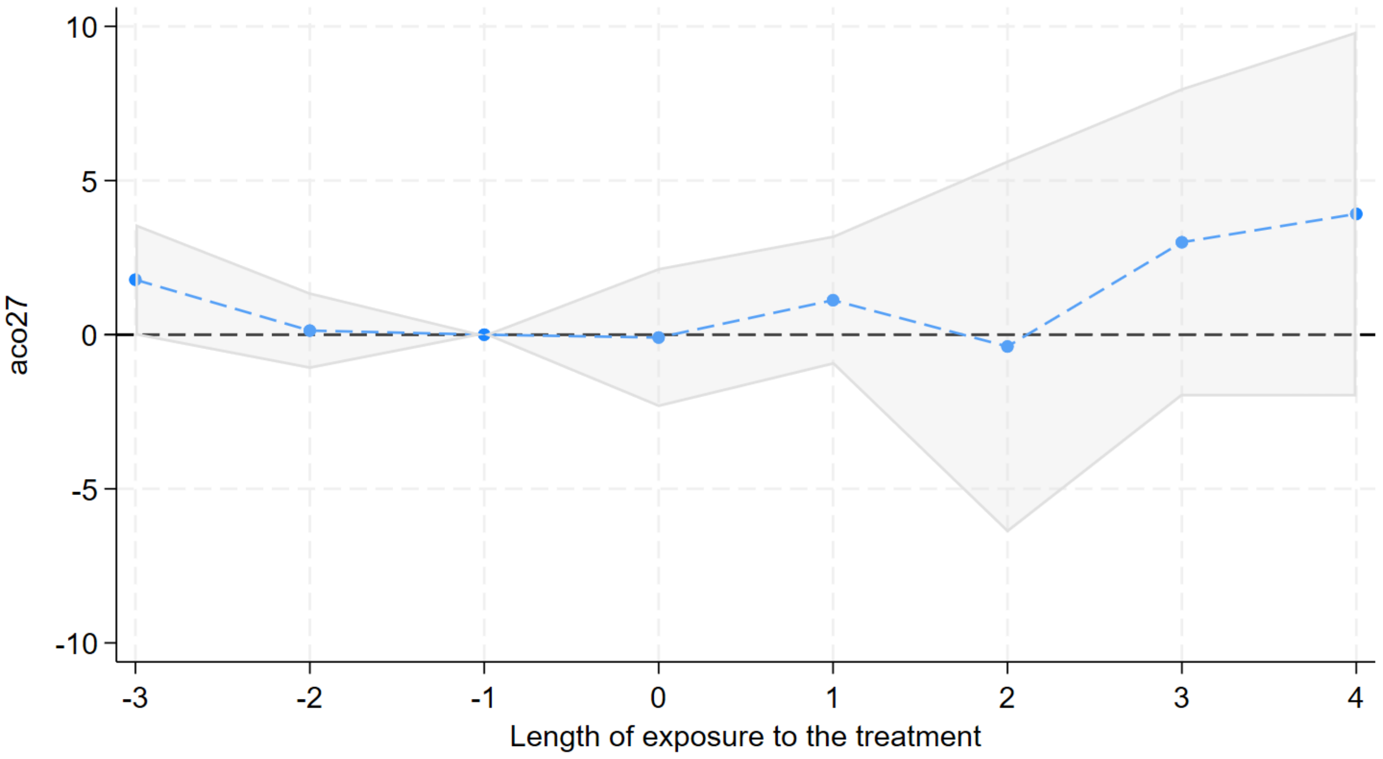

Controlling high blood pressure

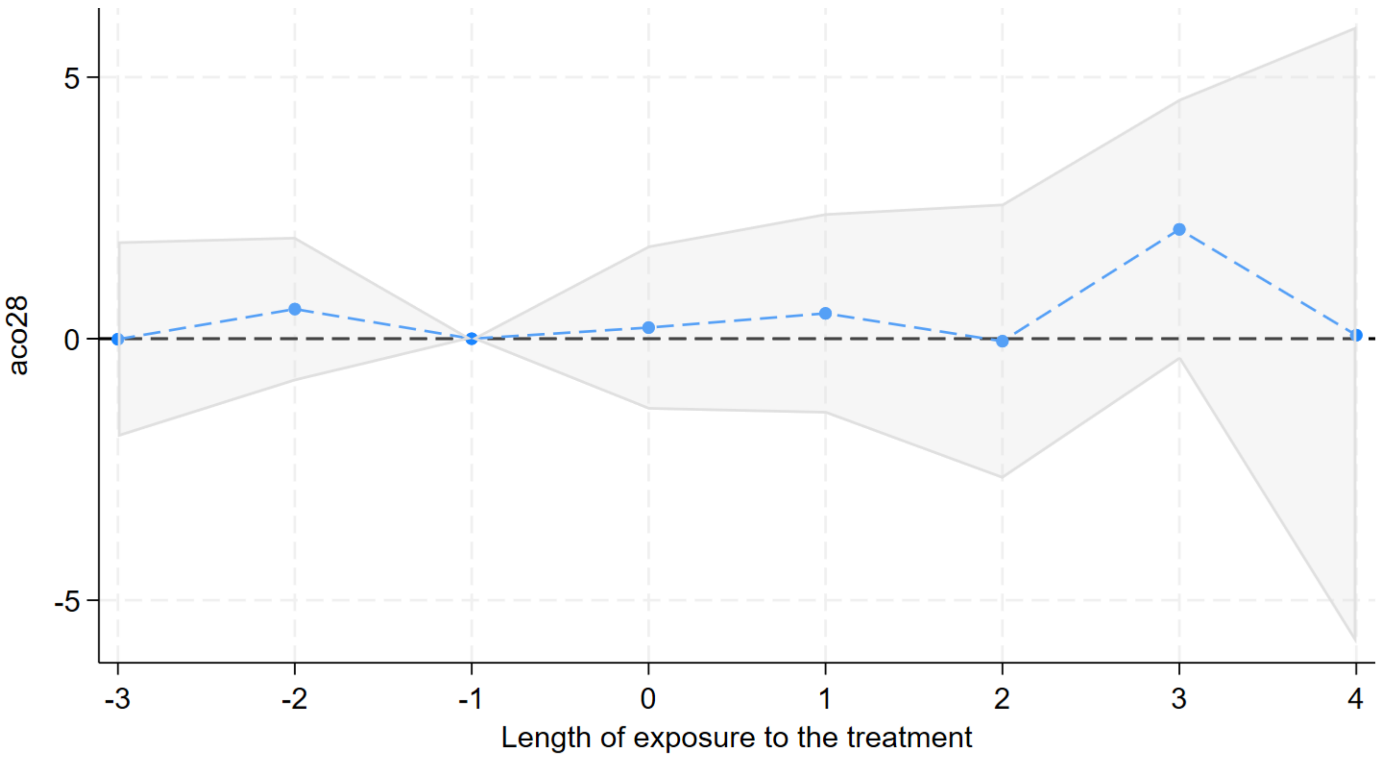

© 2024 Kun L et al. *JAMA Network Open.*
