# Supplementary material for: Federally Qualified Health Centers and Performance of Medicare Accountable Care Organizations
Source: JAMA Netw Open. 2024 Nov 18;7(11):e2445536. doi: 10.1001/jamanetworkopen.2024.45536 (PMC11574694; doi:10.1001/jamanetworkopen.2024.45536)
Supplement: Supplement 2. — Data Sharing Statement [file jamanetwopen-e2445536-s002.pdf]

Data Sharing Statement

Li. Federally Qualified Health Centers and Performance of Medicare Accountable Care Organizations. *JAMA Netw Open*. Published November 18, 2024.
doi:10.1001/jamanetworkopen.2024.45536

Data
Data available: No

Additional Information
Explanation for why data not available: The data is publicly available from the Centers for Medicare and Medicaid Services.
